# Ultrasound-Informed State Estimation of Wrist Tremor Dynamics via Koopman Operator for Personalized Sensory Peripheral Nerve Stimulation

**DOI:** 10.1109/TNSRE.2026.3709711

**Published:** 2026

**Authors:** Xiangming Xue, Vidisha Ganesh, Daniel Roque, Tanya P. Garcia, Xiaoning Jiang, Nitin Sharma

**Affiliations:** Lampe Joint Department of Biomedical Engineering, North Carolina State University, Raleigh, NC 27695 USA.; Lampe Joint Department of Biomedical Engineering, North Carolina State University, Raleigh, NC 27695 USA.; Division of Movement Disorders, Department of Neurology, The University of North Carolina at Chapel Hill, Chapel Hill, NC 27599 USA.; Department of Biostatistics, The University of North Carolina at Chapel Hill, Chapel Hill, NC USA.; Lampe Joint Department of Biomedical Engineering, North Carolina State University, Raleigh, NC 27695 USA; Department of Mechanical and Aerospace Engineering, North Carolina State University, Raleigh, NC 27695 USA.; Lampe Joint Department of Biomedical Engineering, North Carolina State University, Raleigh, NC 27695 USA; Department of Mechanical and Aerospace Engineering, North Carolina State University, Raleigh, NC 27695 USA

**Keywords:** Data-driven modeling, ultrasound sensing, sensory peripheral nerve stimulation, electrical stimulation, tremor suppression, neuromodulation, movement disorders

## Abstract

Tremor is among the most disabling motor symptoms in Parkinson’s disease and essential tremor, yet current pharmacological and surgical therapies remain limited by side effects, invasiveness, and inconsistent efficacy. Sensory peripheral nerve stimulation (sPNS) has emerged as a non-invasive alternative, but its optimization requires computational models that integrate ultrasound-informed state estimation of tremor dynamics and adapt to individual variability. Here, we introduce a data-driven modeling framework that leverages ultrasound sensing within a Koopman operator approach for state estimation to capture wrist tremor dynamics. In a study of six individuals with Parkinsonian or essential tremor, ultrasound-informed models significantly improved accuracy, reducing time-domain prediction error from 38.1% to 21.3% and enhancing frequency-domain measures, including tremor frequency and power ratio. Modal input analysis revealed that ultrasound-derived features from flexor and extensor muscles accounted for > 60% of model sensitivity, while intermuscular latency among sPNS parameters contributed most strongly (31.6%), consistent with the physiological principle of out-of-phase stimulation. The framework also proved resilient to sensor dropout, preserving dominant tremor frequency and temporal features via data-driven interpolation. In a pilot human-validation study involving one Parkinsonian tremor participant and one essential tremor participant, the model-informed protocols selected by the proposed framework produced the strongest suppression among the tested stimulation conditions, achieving mean suppression ratios of 28.3% and 45.1%, respectively. These findings establish ultrasound-informed modeling as a novel state-estimation and modeling strategy for tremor characterization, while also providing initial prospective support for model-guided personalization of sPNS parameters.

## I. INTRODUCTION

Tremor is a common neuromuscular manifestation characterized by involuntary, rhythmic contractions that lead to oscillatory movements of the limbs, head, or voice [1], [2]. It is most prevalent in Parkinson’s disease (PD) and essential tremor (ET), affecting millions worldwide and severely impairing quality of life by disrupting tasks such as writing, eating, and maintaining posture [3], [4], [5].

Existing interventions, such as pharmacological agents [6], deep brain stimulation (DBS) [7], physical or occupational therapy, and robotic exoskeletons, provide only partial and inconsistent benefit. Their limitations arise from medication-related side effects [8], the invasiveness and surgical risks of DBS [9], and the inability of supportive therapies or devices to directly modulate tremor-causing mechanisms. Consequently, there remains a pressing need for non-invasive, adaptable strategies that ensure reliable and sustained suppression.

Sensory peripheral nerve stimulation (sPNS) has gained increasing attention as a promising non-invasive technique for tremor suppression, mainly due to its tolerability and low risk of inducing muscle fatigue. In contrast to functional electrical stimulation (FES), which requires high-intensity currents to activate motor units and may cause fatigue with prolonged use [10], sPNS delivers low-intensity electrical pulses to sensory pathways. This indirect modulation of central motor circuits has been shown to reduce tremor amplitude in select patient populations [11], [12].

Despite promising results, the clinical adoption and consistent efficacy of sPNS remain limited, with outcomes that vary substantially across studies, tasks, and individuals [12], [13]. A key barrier is the lack of systematic, patient-specific parameter-selection frameworks grounded in stimulation-relevant physiological feedback, as stimulation settings are often tuned empirically rather than adapted to individualized neuromuscular dynamics. Intramuscular adaptive stimulation has achieved average suppression of about 32% [14], while fixed or phase-locked out-of-phase approaches have reported reductions of 35–48% [15], [16]. More broadly, reviews indicate acute tremor reductions ranging from 12–90%, depending on task, sensor metric, and stimulation strategy [12], [13]. This heterogeneity motivates a systematic, stimulation-aware modeling framework to characterize nonlinear, time-varying relationships between stimulation inputs and tremor response and support individualized parameter selection.

Clinical evidence for non-invasive sPNS therapies remains mixed across endpoints and study designs. In an acute randomized controlled trial using a commercial wrist-worn device, the treated group did not show a statistically greater improvement than sham on the Archimedes spiral endpoint, despite improvement in other tremor-related subscores [17]. A prospective home-use study reported improvement in multiple clinical measures but also noted device-related adverse events in 18% of participants, without device-related serious adverse events [18]. Importantly, these studies primarily evaluate pre/post therapy effects rather than real-time adaptive parameter modulation during ongoing tremor, leaving open questions about how stimulation settings directly interact with time-varying neuromuscular dynamics.

In parallel, real-time closed-loop sPNS strategies have been explored, but practical sensing and control limitations remain. EMG-based approaches can time stimulation to tremor dynamics, yet often require sequential operation or explicit blanking because stimulation artifacts degrade EMG recordings [15], [16]. More recently, US imaging has been used to trigger afferent stimulation in real time using artifact-free tissue displacement metrics, but current implementations remain predominantly trigger-based and do not provide a stimulation-aware dynamical model for systematic multi-parameter personalization [19]. Collectively, these findings motivate a stimulation-aware, physiology-grounded modeling framework for quantifying subject-specific stimulation–tremor dynamics during intervention and supporting systematic parameter personalization in future data-driven closed-loop studies.

Tremor dynamics arise from multiple interacting factors, including central oscillatory circuits such as basal ganglia and olivocerebellar loops [20], biomechanical resonances of limb segments and reflex pathways [20], neuromuscular variability associated with fatigue [21], and external influences such as mechanical loading or vibration [22]. Because these contributions fluctuate across time and context, static models are inadequate. Adaptive modeling frameworks that integrate physiological feedback in real time are essential to capture tremor behavior during intervention. Previous modeling studies have primarily emphasized classification of tremor types [23], analysis of frequency and phase relationships [16], or mapping of tremorogenic muscle activity [24]. However, the dynamic evolution of tremor under active sPNS input has not been systematically investigated, and addressing this gap forms the central focus of the present study.

Implementing adaptive, data-driven tremor suppression requires reliable and physiologically relevant sensing. Conventional modalities, however, have prominent limitations. Inertial measurement units (IMUs) capture gross limb kinematics and tremor frequency [25], but they cannot resolve muscle-specific activity and often accumulate drift errors that impair long-term accuracy [26]. Electromyography (EMG) provides muscle-specific signals [15], yet is highly susceptible to stimulation-induced artifacts and lacks the ability to separate adjacent or deep muscle layers [27]. As a result, IMU and EMG sensing alone can be challenging for continuous stimulation-aware modeling under sPNS, particularly when artifact-free, muscle-specific tracking is needed during ongoing stimulation.

Ultrasound (US) imaging offers a promising alternative. Unlike IMUs, US measures localized tissue displacement directly within target muscles, and unlike EMG, US is inherently immune to stimulation artifacts, enabling continuous tracking of antagonist muscle dynamics during active stimulation. Recent work has demonstrated that US can quantify muscle coordination and tremor frequency with high specificity [19], [28]. At the same time, ultrasound-based sensing has practical translational limitations in its current form. Many demonstrations rely on commercial ultrasound platforms that are bulky and tethered to external hardware, and robust acquisition typically requires careful probe fixation and stable coupling to minimize relative motion and contact-pressure variability. These constraints can restrict natural movement and limit feasibility for long-duration monitoring and at-home use. Accordingly, translation of ultrasound-informed tremor modeling will benefit from wearable ultrasound hardware and embedded, low-latency processing pipelines that reduce setup burden and tethering while preserving muscle-specific, artifact-robust measurements. Nevertheless, most prior studies have focused on diagnostic or observational use rather than predictive modeling or adaptive control under stimulation. Building on this gap, Xue et al. introduced ultrasound-based data-driven modeling methods for tremor. Using Koopman operator theory, they transformed nonlinear tremor behavior into a linear, higher-dimensional framework amenable to analysis and prediction [29]. Subsequent work extended this framework to a predictive control system, demonstrating the feasibility of adaptive tremor modeling under varying conditions [30]. While these studies provide a strong theoretical foundation, their integration with active sPNS and translation-oriented constraints (e.g., wearable sensing, continuous operation, and real-time adaptation) remains to be investigated.

To address these challenges and advance personalized tremor management, this study introduces a data-driven framework that integrates ultrasound sensing with sPNS parameters. Specifically, three model configurations are developed and assessed: a fixed model using only sPNS inputs, a multisensor model that combines US with inertial data, and an adaptive model with recursive updates. To our knowledge, this is the first work to systematically analyze the influence of individual sPNS parameters within a model-based framework and to use the resulting stimulation-aware model for subject-specific protocol selection. The approach further enables input sensitivity analysis that reveals physiologically relevant stimulation–muscle relationships and demonstrates how US improves modeling accuracy and robustness to sensor dropout, while also supporting model-guided personalization of sPNS settings. In a pilot human-validation study, the model-informed protocols identified for one PD participant and one ET participant produced the strongest tremor suppression among the tested conditions. Together, these contributions establish a foundation for future closed-loop tremor suppression and data-driven personalization studies.

## II. ULTRASOUND-AUGMENTED DATA-DRIVEN MODELING OF TREMOR DYNAMICS UNDER SPNS

This section presents the methodology for developing and validating a data-driven model of tremor dynamics under sPNS. The proposed framework combines Koopman operator theory [31], [32] with experimental data from individuals with tremor, using a lifted linear formulation to represent stimulation-aware tremor dynamics [33]. Ultrasound-derived muscle displacement was incorporated as an artifact-free, muscle-specific physiological input for modeling, and recursive online updates were used to accommodate time-varying tremor behavior [30]. Detailed derivations of the Koopman/EDMD formulation and recursive update procedures are provided in Supplementary Sections S1 and S2, while ultrasound-based displacement estimation and dual-probe compensation procedures are described in Supplementary Section S3.

### A. Experimental Protocol for Multisensor Tremor Recording Under sPNS

This section outlines the protocol for collecting the multi-sensor and sPNS parameter dataset to develop the data-driven models that enable analysis of how specific stimulation parameters influence tremor behavior.

#### 1) Participant Demographics and Selection Criteria:

This study was approved by the Institutional Review Board at North Carolina State University (IRB approval number: 19247) and included four participants with PD and two with ET. Inclusion criteria required individuals to exhibit visible tremor during a grasping task, be between 18 and 90 years old, and have no comorbidities that could affect tremor severity. Tremor severity was assessed using the Essential Tremor Rating Assessment Scale (TETRAS) score [34] for ET and the MDS-Unified Parkinson’s Disease Rating Scale (MDS-UPDRS) Part 3 score [35] for PD. Detailed demographic and clinical information is provided in Supplementary Table S1.

#### 2) Experimental Protocols and Multi-Sensor Data Acquisition:

**Fig. 1.**
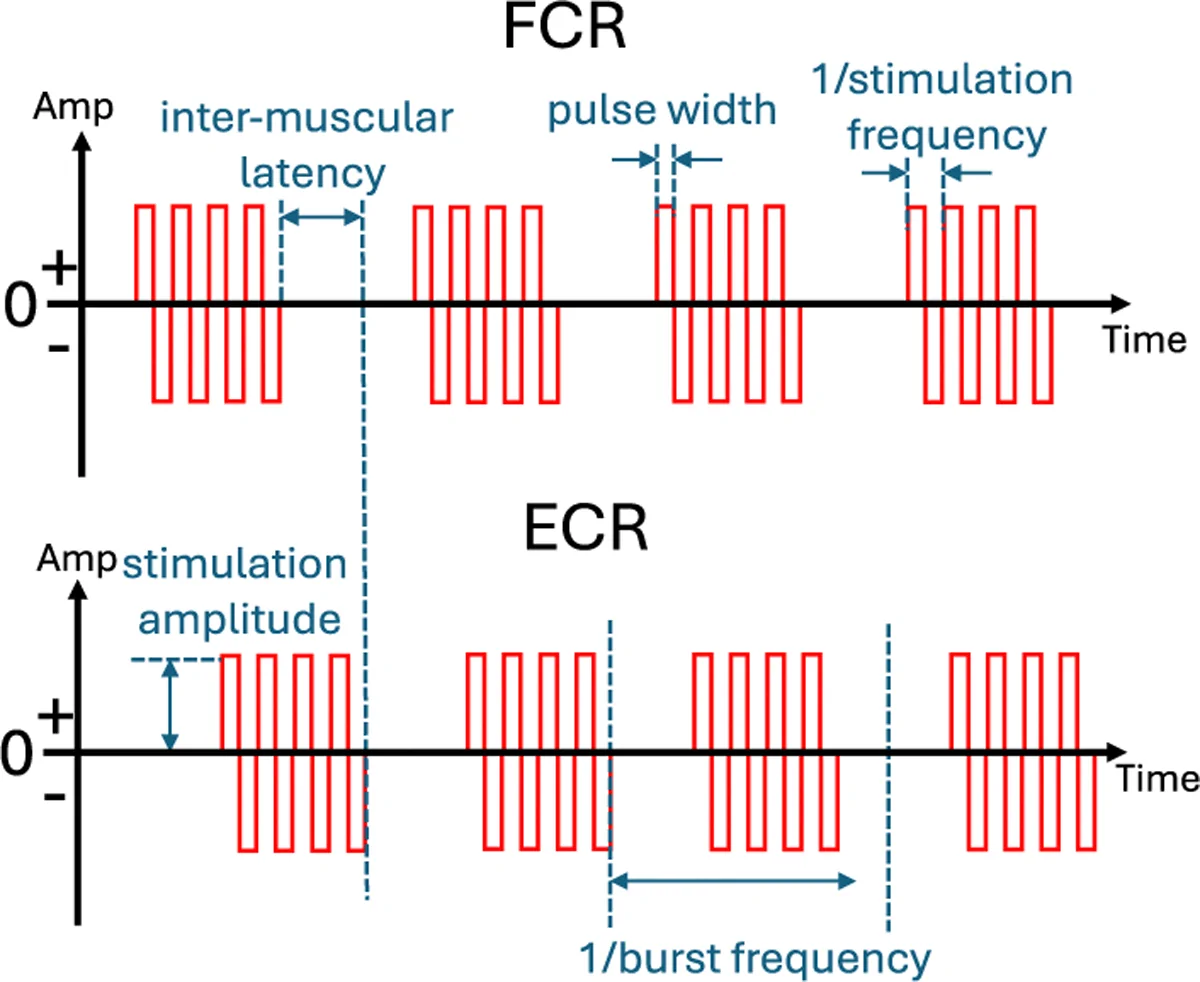
Illustration of Sensory peripheral nerve stimulation (sPNS) parameter configurations applied to the forearm antagonist muscles, flexor carpi radialis (FCR) and extensor carpi radialis (ECR).

The objective of this experiment was to characterize tremor dynamics under varying sPNS conditions by collecting synchronized data from IMUs and US sensors. sPNS parameters, including stimulation amplitude, burst timing, and intermuscular onset latency, were systematically varied to examine their effects on tremor behavior. Definitions and configurations of these stimulation parameters are illustrated in Figure 1. These parameter variations were designed to span a broad input space for stimulation response characterization and data-driven modeling, rather than to optimize stimulation settings for maximal tremor suppression within each session.

**Fig. 2.**
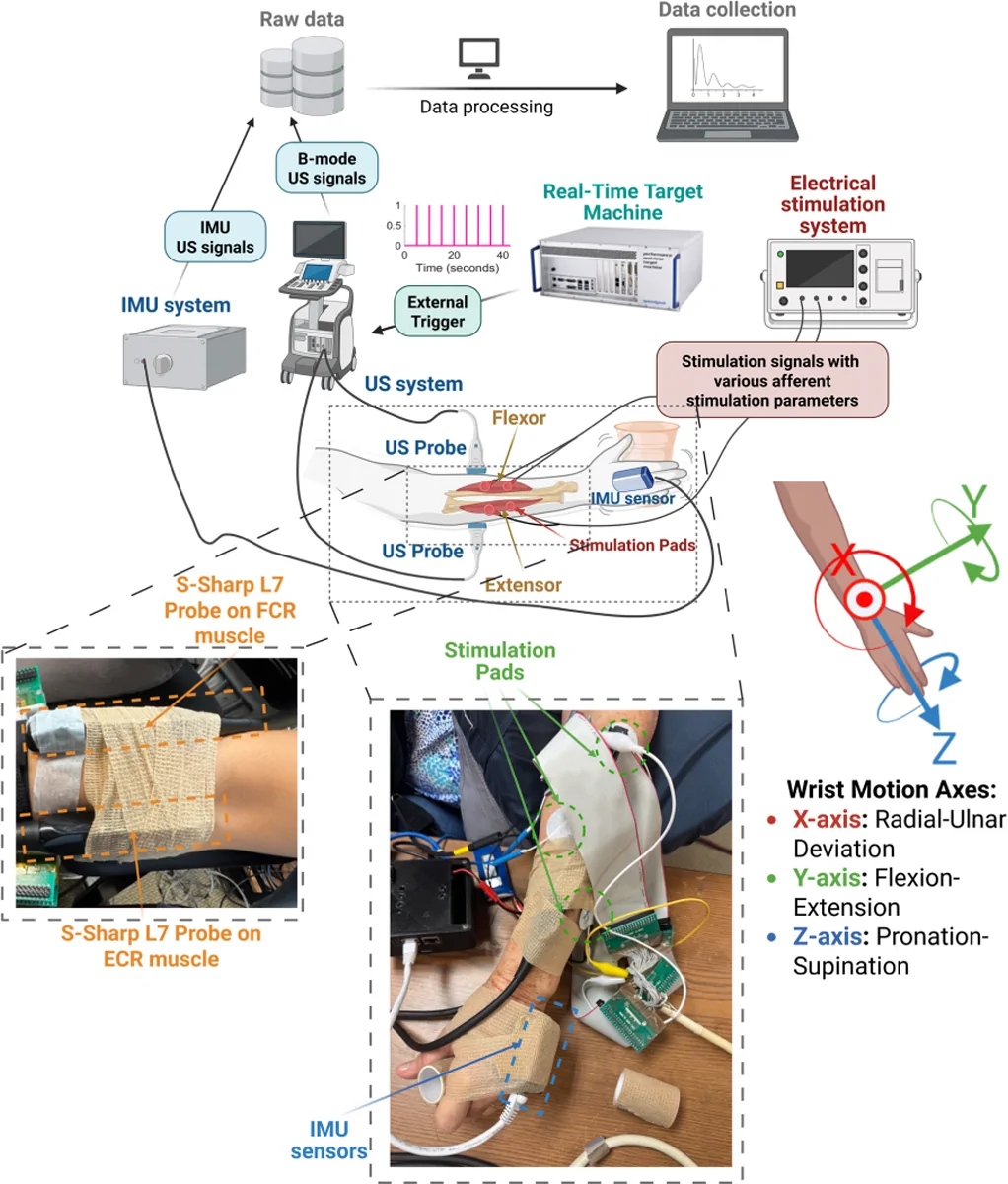
Schematic of the experimental setup for tremor data collection under sPNS.

A multi-sensor setup was used, as shown in Figure 2. Two US probes (L7.0SC Prodigy, S-Sharp, Taiwan) were placed over the FCR and ECR muscles to capture muscle tissue displacement during tremor episodes in response to sPNS. Since each probe collected data only during part of the acquisition cycle, the resulting ultrasound signals for FCR and ECR contained periodic temporal gaps. These discontinuities complicate continuous modeling by obscuring the full temporal evolution of muscle activity. To address this challenge, we implemented a data-driven online update approach capable of reconstructing the missing segments in real time. Full methodological details and supporting results are provided in the Supplementary Materials (see Sections S3). B-mode ultrasound images with ROI and the corresponding ultrasoundderived tissue displacement and wrist kinematics across all participants are provided in Supplementary Section S4–B and Section S4–C, respectively

The IMU (Yost Labs Inc., USA) was attached to the wrist to record kinematic data. Electrical stimulation electrodes (Valutrode, 1.25”, Axelgaard, CA, USA) were placed over both FCR and ECR strategically and connected to a programmable stimulator (Rehastim 2, HASOMED GmbH, Germany). All devices were synchronized via a real-time target machine (Speedgoat, Liebefeld, Switzerland) to ensure precise coordination between data acquisition and stimulation delivery.

During the experiment, participants were seated and instructed to perform a grasp-and-hold task by lifting a cup in response to a visual cue. This grasp-and-hold task was selected to reliably elicit a relatively steady postural tremor state for stimulation response characterization and modeling, while reducing confounds from continuous goal-directed voluntary movement. Spiral drawing is a clinically meaningful functional assessment, but it inherently combines tremor with voluntary motion components (e.g., trajectory planning, speed variation, and visuomotor feedback), which can complicate identification of stimulation effects on tremor in a pilot modeling protocol designed to span a broad parameter space. To ensure repeatable extraction of ultrasound-derived muscle displacement features, the forearm posture was standardized and the probes were secured throughout each trial; spiral-based endpoints will be incorporated in future efficacy-oriented studies alongside standardized clinical and functional outcome measures. The two US probes alternated acquisition at a pulse repetition frequency (PRF) of 1 kHz, with each collecting five frames over a 5 ms interval before switching. The IMU was sampled at 1000 Hz, aligned temporally with US acquisition to allow for direct integration of muscle displacement and wrist motion data.

sPNS parameters were varied systematically throughout the experiment. Stimulation amplitude ranged from 0 to 6 mA, remaining below the motor threshold to elicit afferent responses without triggering muscle contractions. Burst frequency was randomized between 2 and 12 Hz in 2 Hz increments, using a temporal resolution of 0.5 s to match the 4–12 Hz tremor frequency range [36]. Intermuscular latency values were randomized between 0 and 0.25 s, encompassing the 0.1 s latency typically associated with out-of-phase stimulation [15], [16]. Stimulation frequency and pulse width were fixed, with pulse width set at 150 *μ*s based on prior studies showing minimal effect of this parameter on suppression efficacy [11], [37], and frequency selected from optimal and sub-optimal values identified in previous work [19]. During each session, participants were monitored for comfort and skin condition at the electrode and contact sites, with rest provided as needed and immediate discontinuation if any undue discomfort or skin irritation was observed, consistent with IRB-approved risk mitigation procedures.

### B. Construction of Data-Driven Models for Tremor Dynamics

This subsection describes the data-driven model formulations used to characterize tremor dynamics under sPNS. As described in Section II–A.2, stimulation parameters were systematically varied to probe their influence on tremor behavior. The modeling framework serves two key objectives: (1) to quantify how different sPNS parameters affect tremor dynamics as captured by both IMU and US sensing, and (2) to enable real-time model adaptation for robust tracking of tremor responses under varying stimulation conditions. Additional signal processing details (Section S5–A) and observablefunction selection procedures (Section S5–B) are provided in the Supplementary Material.

#### 1) Comparison of Data-Driven Model Variants for Tremor Dynamics:

To evaluate the influence of sensor integration and model adaptability on tremor modeling accuracy, we developed three categories of data-driven models under sPNS. All models aim to predict wrist angle kinematics *x*_*k*_ along three axes (x, y, z) as defined in Figure 2, with variations in their sensor input configurations and update mechanisms. In this proof-of-concept study, ultrasound imaging was performed over the FCR/ECR muscle group, which primarily contributes to wrist flexion–extension, a commonly dominant tremor axis. The tissue displacement features were extracted from the 2D B-mode imaging plane and reflect local muscle motion within the imaging slice (axial/lateral components), rather than a full three-dimensional displacement vector of the wrist. These ultrasound-derived features were incorporated into the Koopman/EDMD state-space model as muscle-specific physiological states that complement the joint kinematics, facilitating identification of stimulation–muscle–kinematic coupling.

##### a) Model 1: fixed-parameter sPNS-only model:

This baseline model incorporates only sPNS as input and uses fixed system matrices. The model is defined as:

(1) $$x_{k+1}=Ax_{k}+Bu_{sPNS},$$

where $x_{k}\in R^{3}$ denotes the wrist angle states, and $u_{sPNS}\in R^{4}$ includes stimulation amplitude to FCR $\left(u_{{sPNS}_{FCR}}\right)$, stimulation amplitude to ECR $\left(u_{{\text{sPNS}}_{ECR}}\right)$, burst frequency $\left(u_{{\text{sPNS}}_{\mathrm{brt}}}\right)$, and intermuscular latency between FCR and ECR $\left(u_{{\text{sPNS}}_{\mathrm{lat}}}\right)$. A is a fixed system matrix, and B is a fixed input matrix for sPNS. This model does not include US sensor input.

##### b) Model 2: fixed-parameter sPNS + ultrasound model:

To assess the effect of integrating muscle-specific sensing, this model extends the input vector to include ultrasound-derived tissue displacement. The model is expressed as:

(2) $$x_{k+1}=Ax_{k}+Bu_{sPNS}+Hu_{US},$$

where $u_{US}\in R^{2}$ consists of displacement from the FCR muscle $\left(u_{{US}_{FCR}}\right)$ and from the ECR muscle $\left(u_{{US}_{ECR}}\right)$. H is the ultrasound input matrix identified from data during model training (i.e., a data-driven mapping from $u_{US}$ into the learned linear dynamics), and the remaining parameters are the same as in Eq (1). For Model 2, H is held fixed during prediction after training; for Model 3, H is updated online together with A and B via the RLS-based recursive update.

##### c) Model 3: adaptive sPNS + ultrasound model with online parameter updates:

The third model introduces adaptability through real-time parameter updates using a recursive least squares (RLS) strategy. It accounts for dynamic variations in tremor characteristics by continuously refining the model at each time step:

(3) $$x_{k+1}={\overset{ˆ}{A}}_{k}x_{k}+{\overset{ˆ}{B}}_{k}u_{sPNS}+{\overset{ˆ}{H}}_{k}u_{US},$$

where ${\overset{ˆ}{A}}_{k}$, ${\overset{ˆ}{B}}_{k}$, and ${\overset{ˆ}{H}}_{k}$ represent the adaptive system matrices, i.e., recursively updated estimates at time step k based on the incoming tremor state and input data.

#### 2) Model Training and Prediction Procedure:

All three models were trained and evaluated using a consistent procedure. The dataset was divided into equal-length training and testing segments. Model accuracy was assessed based on how well each configuration predicted wrist angle kinematics from the learned dynamics during the training phase.

For Model 1 (Eq (1)), training involved estimating the state transition matrix *A* and sPNS input matrix *B* using wrist angles *x*_*k*_ and sPNS parameters *u*_sPNS_. During prediction, future wrist angles were computed solely from sPNS inputs using the fixed parameters *A* and *B*.

For Model 2 (Eq (2)), the training stage extended this formulation to include US inputs *u*_US_, allowing identification of the additional input matrix *H*. Prediction used both *u*_sPNS_ and *u*_US_ as inputs to generate wrist kinematics based on the fixed matrices *A*, *B*, and *H*.

For Model 3 (Eq (3)), initial training mirrored the process in Model 2, yielding starting estimates for $\overset{ˆ}{A}$, $\overset{ˆ}{B}$, and $\overset{ˆ}{H}$. During the prediction phase, these parameters were adaptively updated in real time using a recursive least squares (RLS) approach, continuously incorporating new measurements of wrist angles, sPNS parameters, and US inputs.

The performance of all models during the prediction phase is evaluated in Section III by comparing predicted wrist angles to ground truth data from the test segment.

### C. Evaluation of Model Accuracy and Tremor Frequency: Metrics and Outcomes

To assess the performance of our data-driven tremor models under sPNS, we employed both time-domain and frequency-domain evaluation metrics. These metrics capture the fidelity of modeled wrist kinematics and provide physiologically relevant insights into tremor behavior.

#### 1) Time-Domain Accuracy: nRMSE Metric and Predictive Performance:

We evaluated the reconstruction quality of the predicted wrist angles using the nRMSE, which quantifies the deviation between the predicted output and actual sensor measurements. The nRMSE is defined as:

(4) $$nRMSE=\sqrt{\frac{1}{N}\sum_{i=1}^{N}{\left(\frac{y_{\text{actual},i}\;-\;y_{\text{predicted},i}}{y_{max}\;-\;y_{min}}\right)}^{2}},$$

where $y_{\text{actual},i}$ and $y_{\text{predicted},i}$ are the actual and predicted values for the *i*-th data point, respectively, and $y_{max}$ and $y_{min}$ denote the range of the actual signal. This metric provides a normalized indication of temporal prediction accuracy across models.

**Fig. 3.**
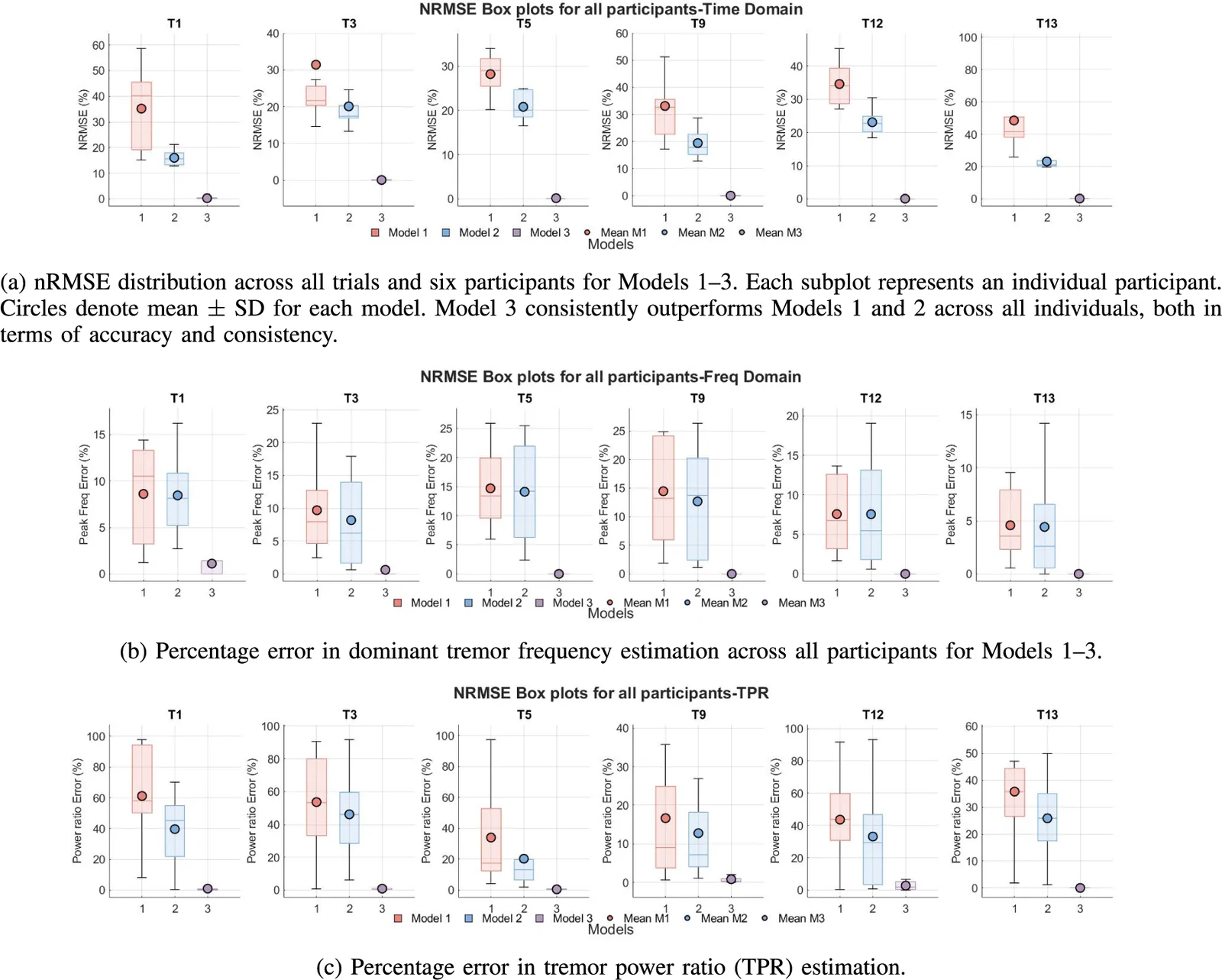
Comparative model performance across participants: (a) Time-domain nRMSE, (b) dominant tremor frequency error, and (c) tremor power ratio (TPR) error.

Figure 3a summarizes the distribution of trial-level nRMSEs for all six participants across the three models: Model 1 (sPNS-only), Model 2 (sPNS + US), and Model 3 (sPNS + US with online parameter updates).

Model 3 consistently achieved the lowest nRMSE across all participants, with participant-level averages ranging from 0.04% to 0.29%. Model 2 exhibited moderate improvements over Model 1, with mean nRMSEs between 16.0% and 23.2%, compared to 28.2% to 48.5% for Model 1. Importantly, the addition of US sensing in Model 2 not only reduced the average error but also lowered the trial-to-trial variability, as indicated by smaller standard deviations. This highlights the value of integrating physiological sensing to improve model robustness and precision in tremor prediction. Detailed numerical values for the time- and frequency-domain performance metrics across all participants are provided in Supplementary Table SII. A representative time-domain comparison is provided in Supplementary Fig. S8.

While prediction accuracy is not itself a clinical endpoint, continuous wrist kinematic trajectories provide an objective proxy for tremor severity during stimulation. Prior studies have shown that motion-sensor–derived tremor features can support automated neuromodulation optimization and correlate with tremor outcomes after DBS [38], [39]. These precedents motivate our use of wrist-angle trajectory prediction as a continuously trackable modeling output that can be related to functional and clinical outcomes in future studies.

#### 2) Frequency-Domain Accuracy: Dominant Frequency Metric and Spectral Agreement:

To evaluate how well the models preserved the spectral characteristics of tremor, we extracted the dominant tremor frequency, defined as the frequency at which the signal’s power spectral density reaches its maximum. This was determined by computing the Fourier Transform X(f) of the signal and identifying the frequency f that maximizes its magnitude:

(5) $$f_{\text{dominant}}=arg\;\underset{f}{max}\;|X(f)|.$$

This frequency reflects the most prominent oscillatory component in the tremor and is widely used in clinical characterization of tremor types [40]. Clinically, tremor frequency is a core quantitative descriptor used in neurological and electrophysiological assessment to characterize tremor syndromes and support phenotyping and differential diagnosis [41]. Its clinically interpretable range across disorders and tasks (e.g., ET often 4–12 Hz and PD commonly 4–7 Hz) also makes dominant-frequency estimates useful for quantitative classification, longitudinal tracking, and instrumented tremor evaluation [42], [43].

We also quantified the concentration of signal power within the typical tremor frequency band (4–12 Hz) relative to the total power within a broader physiological bandwidth (0–20 Hz). This tremor power ratio was computed as:

(6) $$\text{Tremor}\;\text{Power}\;\text{Ratio}=\frac{\sum_{f=4}^{f=12}|X(f){|}^{2}}{\sum_{f=0}^{f=20}|X(f){|}^{2}}\text{,}$$

where $|X(f){|}^{2}$ denotes the power spectral density at frequency f. This metric highlights the proportion of motion attributable to tremor-like behavior and is especially useful for distinguishing tremor from voluntary or background movements [44]. Beyond dominant frequency, tremor-band power distribution metrics are widely used as objective descriptors of tremor severity and treatment response [45], [46]. Accordingly, the tremor power ratio provides a compact summary of how strongly signal energy is concentrated within the canonical tremor band, complementing frequency-based evaluation [43].

We quantified the percentage error in two tremor-related spectral features: (1) dominant tremor frequency, and (2) tremor power ratio (TPR), which represents the proportion of signal energy within the 4–12 Hz tremor band. The error metric was computed as

$$\text{Error}=\left|\frac{\text{Predicted}\;-\;\text{Ground}\;\text{Truth}}{\text{Ground}\;\text{Truth}}\right|\times 100\%.$$

Figure 3b shows the percentage error in dominant frequency across all six participants. Compared to Model 1 (mean errors ranging from 4.6% to 14.7%) and Model 2 (4.4% to 14.1%), Model 3 achieved near-zero frequency estimation error in all patients. The standard deviations for Model 3 were also consistently lower, highlighting improved robustness. This supports the value of online updates in tracking time-varying spectral dynamics.

Figure 3c presents the percentage error in TPR. Model 1 exhibited the highest TPR error across all participants (e.g., 61.2% for T1 and 53.7% for T3), while Model 2 reduced errors substantially (e.g., 39.7% for T1 and 46.2% for T3). However, Model 3 outperformed both, achieving errors under 3% across all participants. These improvements suggest that real-time modeling not only preserves frequency information but also better captures signal amplitude characteristics over the tremor band. A representative frequency-domain comparison is provided in Supplementary Fig. S9.

These findings confirm the importance of US integration (Model 2) for enhancing frequency resolution over afferent-only modeling (Model 1), and further demonstrate that real-time recursive modeling (Model 3) delivers the most accurate frequency-domain performance across all patients.

### D. Statistical Evaluation of Model Performance

To compare the three modeling approaches across participants, we performed within-subject statistical analyses on patient-level mean nRMSE values. Because the data were non-Gaussian and repeated across matched subjects, we used the Friedman test, followed by Wilcoxon signed-rank post hoc comparisons with Holm–Bonferroni correction. The Friedman test indicated a significant overall effect of model type on error (*χ*^2^(2) = 12.0, *p* = 0.0025). Post hoc comparisons yielded raw *p*-values of 0.0313 for all model pairs, which increased to 0.0938 after Holm–Bonferroni correction. Thus, while the overall test supports differences among the three models, specific pairwise differences could not be conclusively resolved given the small sample size (*n* = 6).

## III. MODAL DECOMPOSITION AND TREMOR CHARACTERIZATION FROM DATA-DRIVEN MODELS

Beyond reconstructing tremor wrist motion, data-driven models also provide insight into the underlying neuromechanical dynamics through their internal structure. This section examines how Koopman-based models can be used for physiological interpretation and input sensitivity analysis by first assessing the contribution of sPNS and US inputs and then identifying which wrist kinematic states dominate tremor expression. While tremor features can be computed directly from IMU wrist-angle and ultrasound displacement time series, such analyses remain primarily descriptive. In contrast, the stimulation-aware Koopman/EDMD model captures temporal dynamics and stimulation coupling, enabling model-based sensitivity analysis, prediction under different stimulation settings, and gap-resilient state estimation when measurements are intermittent.

### A. Input Modal Contributions of sPNS and Ultrasound Sensing

#### 1) Computational Implementation:

Input dominance of afferent stimulation (sPNS) and ultrasound features was quantified using a controllability-Gramian–based subspace analysis; full computational details are provided in the Supplementary Material, Section S8.

#### 2) Method Application:

The analysis was applied to three configurations derived from Model 2: an sPNS-only case using *A* and *B*, a US-only case using *A* and *H*, and a combined case using *A* together with [*B H*]. These configurations were used to isolate the relative contributions of stimulation inputs, ultrasound-derived muscle displacement inputs, and their joint effects on the learned dynamics.

#### 3) Results:

**Fig. 4.**
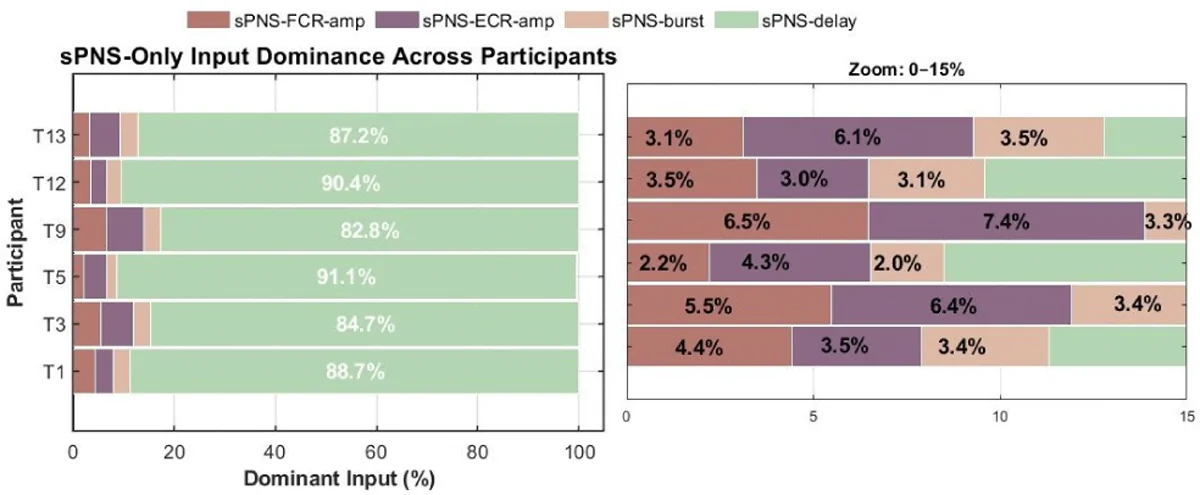
Dominance of sPNS inputs across all participants. The sPNS-delay parameter clearly dominates over amplitudes and burst frequency.

Figure 4 illustrates input dominance percentages for the sPNS-only model, highlighting that intermuscular stimulation latency (sPNS-delay) overwhelmingly dominated (82.8–91.1%) across all participants. Other sPNS parameters, FCR and ECR stimulation amplitudes, as well as burst frequency, collectively accounted for less than 20% dominance, indicating that the stimulation delay is the most influential parameter within the sPNS input subset.

Additional US-only input-dominance results are provided in Supplementary Fig. S11. In the US-only configuration, participants displayed substantial variability in dominance distribution between the FCR and ECR displacement inputs: the FCR input dominated for participants T1, T3, and T12 (48–69%), whereas the ECR input showed stronger dominance for participants T5, T9, and T13 (52–72%). This result underscores the individualized nature of muscle-specific tremor expression and motivates subject-specific interpretation of ultrasound sensing locations.

The combined sPNS+US input-dominance results are shown in Supplementary Fig. S12. In this configuration, the two US inputs (US-FCR and US-ECR) exhibited substantially larger dominance percentages (33.96 ± 16.68% and 38.06 ± 20.82%, respectively) than most sPNS parameters, such as stimulation amplitudes and burst frequency (all < 2%). However, the sPNS-delay parameter retained a substantial contribution (25.42 ± 13.34%), indicating that intermuscular timing remained highly influential even in the presence of direct physiological measurements.

Taken together, these analyses show that ultrasound-derived muscle displacement inputs contribute strongly to the learned tremor dynamics, while intermuscular delay remains the most influential parameter within the sPNS subset. This result motivates the dominance-guided prioritization strategy used in the subsequent protocol-selection framework.

### B. Identification of Dominant Tremor States

#### 1) Computational Implementation:

To characterize which joint-level states most strongly sustain tremor, we analyzed the eigenstructure of the learned linear predictors in the lifted space and mapped dominant modes back to wrist kinematics. The full computational procedure is provided in Supplementary Section S9.

#### 2) Method Application:

The dominant tremor state analysis was applied to Model 2, which incorporates both sPNS and US-derived displacement inputs. Modal contributions for each angular state (*θ*_*x*_, *θ*_*y*_, and *θ*_*z*_) were computed from the learned model and compared against signal-based contributions derived from the corresponding ground-truth wrist-angle trajectories.

#### 3) Results:

Across participants, prediction errors in state dominance were comparable for *θ*_*x*_, *θ*_*y*_, and *θ*_*z*_, indicating no strong overall axis-specific bias in the learned model. However, subject-specific variability was observed, suggesting that the relative contribution of individual wrist-angle states to tremor expression differs across participants. Detailed state-dominance values are provided in Supplementary Table SIII.

## IV. FRAMEWORK FOR MODEL-GUIDED SUBJECT-SPECIFIC SPNS PARAMETER SELECTION

The analyses in Section III established that the learned stimulation-aware model provides not only predictive accuracy, but also control-relevant information about the relative influence of individual stimulation inputs. Building on these insights, this section presents a general framework for model-guided, subject-specific sPNS parameter selection. The framework uses model-derived parameter prioritization to define the protocol search space, uses subject-specific modeling to evaluate candidate stimulation protocols computationally, and finally prospectively validates the selected protocols in human experiments.

### A. Extracting Control-Relevant Stimulation Priorities From the Learned Model

Building on the input-dominance analysis in Section III–A, we used the learned stimulation-aware model as a practical guide for subject-specific protocol search. Rather than treating all stimulation parameters equally, the model-derived dominance structure was used to prioritize which protocol dimensions should be searched more finely and which could be fixed or sampled more coarsely. This provides a general design principle for model-guided sPNS parameter selection, while allowing the priority structure to vary across subjects or cohorts.

In the present cohort, the sPNS-only dominance analysis showed that intermuscular delay was consistently the most influential stimulation parameter, accounting for 82.8–91.1% of the sPNS-only input dominance across participants (Fig. 4). By contrast, stimulation amplitudes and burst frequency contributed substantially less within the sPNS subset. Accordingly, for protocol search, stimulation amplitude was fixed at a practical reference level of 4 mA, while burst frequency *f*_*b*_ and intermuscular onset latency τ_delay_ were searched over refined candidate sets within the experimentally explored and modeled range. Specifically, *f*_*b*_ was searched over {2, 3, 4, …, 12} Hz, and τ_delay_ was searched over {0, 0.01, 0.02, …, 0.25} s. In this way, the most control-relevant dimension was assigned the finest search resolution, while lower-priority dimensions were fixed or searched more coarsely to reduce combinatorial complexity. More generally, however, the same framework can prioritize other stimulation dimensions if they emerge as more influential in future subjects.

### B. Subject-Specific Model-Based Protocol Search and Ranking

For protocol search and ranking, we used a subject-specific sPNS-only Koopman/EDMD prediction model constructed from each participant’s historical stimulation-response trials. Specifically, the prediction model followed the fixed-parameter sPNS-only formulation introduced as Model 1 in Section II–B.1, while model refinement followed the recursive online-update principle introduced in Model 3. This hybrid formulation was adopted because candidate stimulation settings must be evaluated prospectively under hypothetical sPNS inputs, whereas future ultrasound trajectories are not available *a priori* for untested protocols. Thus, the protocol-selection framework retained the wrist-angle state representation of the main modeling framework, but used sPNS-only inputs for short-horizon forward prediction.

Using the dominance-guided search space defined in Section IV–A, we next used the subject-specific model to test the candidate stimulation protocols computationally. In this way, the protocol search was performed on the model rather than directly on the participant, allowing many candidate combinations to be evaluated efficiently before prospective testing.

Candidate protocols were then ranked according to their predicted suppression performance, and the top-ranked protocol was selected as the model-informed patient-specific stimulation protocol. Thus, the learned model served as a practical protocol-selection engine: it reduced the need for exhaustive empirical testing on the participant while providing a subject-specific ranking of candidate stimulation settings. Supplementary material Section S10 provides additional details on the ranking output.

### C. Human Validation of Model-Informed Stimulation Protocols

To prospectively validate the proposed model-guided protocol-selection framework, we conducted a pilot human study in two representative participants, including one participant with Parkinsonian tremor (T9) and one participant with essential tremor (T12). Using the procedure described in Section IV–B, the top-ranked model-informed protocols were identified as (*A*_FCR_, *A*_ECR_, *f*_*b*_, τ_delay_) = (4, 4, 2, 0.10) for T9 and (4, 4, 2, 0.05) for T12.

The prospective validation followed the object-holding paradigm described in Section II–A. Each trial lasted 60 s, with the first 25 s serving as the initial no-stimulation window and the remaining 35 s serving as the stimulation (or pseudo-stimulation) window; the only exception was C0, in which no stimulation was applied throughout the trial. Each condition was repeated three times. Four conditions were tested: C0, no-stimulation baseline; C1, a general-good reference protocol with continuous 4 mA stimulation and no burst-frequency or delay modulation; C2, the model-informed optimal protocol selected from Section IV–B; and C3, a delay-perturbed comparator, with τ_delay_ = 0.08 for T9 and τ_delay_ = 0.04 for T12 while keeping the remaining stimulation parameters unchanged.

For each trial, the three-axis angular-velocity magnitude was then computed as

$$w_{mag}(t)=\sqrt{\omega_{x}(t{)}^{2}+\omega_{y}(t{)}^{2}+\omega_{z}(t{)}^{2}}.$$

**Fig. 5.**
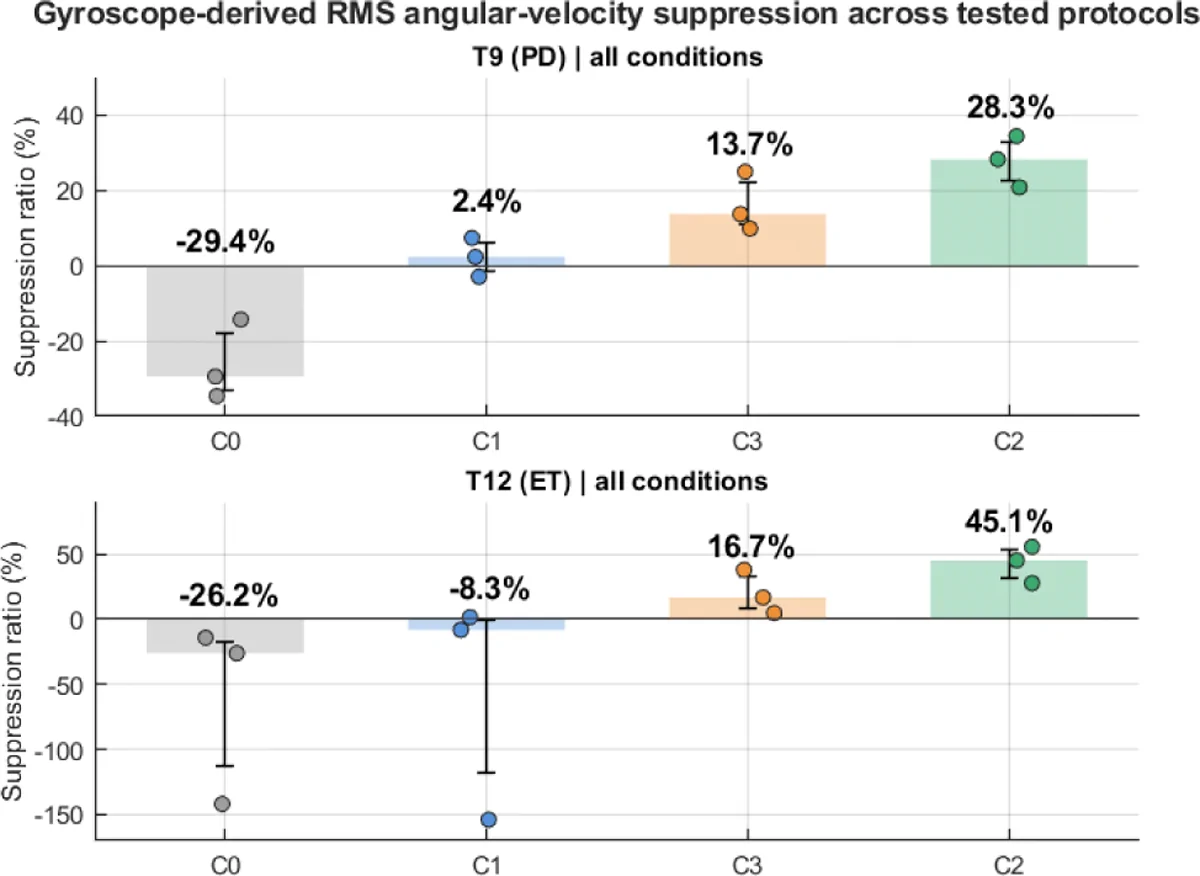
Gyroscope-derived RMS angular-velocity suppression ratios across tested stimulation conditions in the two pilot human-validation participants: T9 (PD) and T12 (ET).

For the initial window (0–25 s) and the later protocol window (25–60 s), suppression was quantified using the RMS of $w_{\text{mag}}(t)$:

$$\text{Suppression}\;\text{Ratio}=1-\frac{{RMS}_{\text{protocol}}}{{RMS}_{\text{initial}}},$$

where ${RMS}_{\text{initial}}$ denotes the RMS of the three-axis angular-velocity magnitude in the initial no-stimulation window and ${RMS}_{\text{protocol}}$ denotes the corresponding RMS in the later protocol window. Positive values therefore indicate tremor suppression, whereas negative values indicate worsening relative to the initial window. Figure 5 summarizes the resulting suppression ratios across tested protocols.

As summarized in Fig. 5, the model-informed protocol (C2) produced the strongest average suppression in both participants. For T9, mean suppression improved from 2.4% under C1 and 13.7% under C3 to 28.3% under C2. For T12, C1 remained ineffective on average (−8.3%), whereas C3 yielded moderate suppression (16.7%) and C2 produced the strongest suppression (45.1%). In contrast, the no-stimulation baseline condition C0 showed negative suppression ratios in both participants (−29.4% for T9 and −26.2% for T12). Representative trial-level comparisons are shown in Supplementary Fig. S13.

These pilot results support the proposed model-guided framework. In both participants, the model-informed protocol outperformed the general-good reference protocol, and perturbing the prescribed delay reduced suppression relative to C2. Together, these findings indicate that the learned model can provide a practically useful subject-specific ranking of candidate sPNS settings.

## V. DISCUSSION

### A. Advancing Personalized Tremor Modeling: Comparison With Prior Approaches

Prior research highlights the critical importance of personalization in tremor modeling due to significant inter-subject variability and nonlinear tremor dynamics. Western et al. [47] demonstrated that personalized tremor profiling enhances the differentiation of clinically relevant tremor changes from confounding factors. Similarly, Farhani et al. [48] emphasized the need for individualized modeling to distinguish voluntary movements from tremor effectively. However, existing approaches primarily rely on static patient-specific profiles or offline modeling techniques, which limit their adaptability and effectiveness in dynamic, real-time tremor management scenarios.

Our study addresses these limitations by employing a data-driven modeling approach capable of continuously adapting to patient-specific tremor dynamics. The integration of US sensing with sPNS parameters allows our model to capture individualized muscle-specific behaviors in real-time. Unlike surface EMG-based methods, which suffer from electrical interference and saturation during stimulation [49], [50], [51], [52], our US-based sensing provides robust and artifact-free measurements of muscle displacement. Moreover, while IMU-based assessments are commonly used for their practicality [53], [54], they inherently lack muscle specificity and fail to offer insights into muscle-level interactions critical for targeted intervention.

Quantitatively, our ultrasound-integrated approach significantly improves modeling fidelity compared to existing tremor prediction methods. Prior studies have reported typical prediction horizons of only 0.25 to 2 seconds, with nRMSE ranging from 8% to 20% [55], [56]. In contrast, our fixed-parameter ultrasound-enhanced model (Model 2) achieves a comparable time-domain nRMSE of 20.43% and a dominant frequency estimation error of 9.57%, with a notable advancement in extending the prediction horizon to approximately 20 seconds. Further substantial improvement is observed with our adaptive online-update model (Model 3), achieving dramatically reduced errors of 0.48% (time domain) and 0.26% (frequency domain), demonstrating superior prediction accuracy. Collectively, these results demonstrate the modeling advantages of our personalized, adaptive ultrasound-based approach over existing frameworks, and the pilot human validation further supports its translational relevance for future real-time implementations and data-driven closed-loop studies.

### B. Physiological Interpretability and Control-Relevant Insights

Our data-driven modeling approach not only improves predictive accuracy but also provides physiologically grounded, control-relevant insights into tremor mechanisms that can inform personalization and hypothesis generation for future tremor suppression studies.

The input sensitivity analysis revealed a clear dominance of ultrasound-based tissue displacement inputs, accounting for over 60% of total model sensitivity. Specifically, muscle displacement data from the flexor carpi radialis (FCR) and extensor carpi radialis (ECR) contributed 34.3% and 29.5%, respectively, highlighting the importance of muscle-level sensing for tremor modeling. This result further emphasizes the advantage of US over surface EMG or IMU, which do not provide equally robust artifact-free tracking of muscle-specific activity during stimulation.

Among sPNS parameters, intermuscular latency emerged as the most influential input (31.6%), exceeding stimulation amplitude and burst frequency. This finding is consistent with prior work on out-of-phase stimulation, in which asynchronous timing between antagonist muscles is used to modulate tremor [11], [15]. In contrast to approaches that require intermittent sensing or artifact-mitigation strategies, our ultrasound-based framework supports continuous stimulation-aware muscle-state tracking. Importantly, the pilot human validation demonstrates that these stimulation-aware muscle-state estimates can be used prospectively to guide subject-specific protocol selection, providing an initial pathway toward future closed-loop and endpoint-driven personalization studies.

We also observed consistently greater model sensitivity in the FCR than in the ECR, in agreement with prior clinical observations. Pascual-Valdunciel et al. [57] reported higher tremor-frequency EMG activity in the FCR than in the ECR during postural tasks in ET, which supports our finding that the FCR often acts as the more tremor-dominant muscle. This suggests that future tremor-suppression strategies may benefit from prioritizing interventions targeting the FCR when subject-specific data support such dominance.

Lastly, dominant-state analysis provides insight into how individual wrist-angle components contribute to tremor persistence and decay. Unlike IMU signals alone, which capture gross kinematics without differentiating axis-specific influences, the learned state-space model can identify which motion states dominate tremor behavior through its eigenstructure. While many tremor descriptors can be computed directly from measured IMU and ultrasound signals, the stimulation-aware dynamical representation adds system-level information by linking stimulation inputs, muscle states, and tremor kinematics within a single interpretable framework. Taken together, these analyses show that the proposed model offers not only improved prediction, but also a structured basis for mechanistic interpretation and future personalized control design, with the added pilot human validation providing an initial prospective step in that direction.

### C. Implications for Personalization and Parameter Adaptation

This study is not a definitive efficacy trial and does not claim superiority on standardized clinical endpoints. However, the pilot human validation provides prospective evidence that the proposed stimulation-aware modeling framework can guide subject-specific sPNS parameter selection and improve tremor suppression relative to generic or perturbed alternatives. Rather than replacing direct measurement-based tremor assessment, the framework complements it by providing an explicit stimulation-aware dynamical representation that can be interrogated, simulated, and used to narrow the parameter search space for future personalization and closed-loop studies.

A key practical implication is that patient-specific sPNS prescription should be treated as a low-query optimization problem rather than an exhaustive empirical search. In the proposed framework, parameter prioritization is not heuristic, but derived from model-based input-dominance analysis, which identifies the stimulation dimensions most likely to influence tremor dynamics for a given subject. In the present cohort, this analysis highlighted intermuscular delay as the most influential sPNS parameter, motivating finer delay exploration during protocol search. The pilot human-validation results further support this interpretation: in both tested participants, the model-informed protocol outperformed the general-good reference condition, and perturbing only the prescribed delay reduced suppression relative to the selected protocol. Together, these findings suggest that the learned model can serve as a practical tool for subject-specific sPNS parameter ranking in future endpoint-driven studies.

### D. Limitations and Future Directions

A primary limitation is that this study was not designed as a definitive clinical efficacy trial of sPNS therapy (e.g., superiority versus sham on standardized clinical endpoints such as spiral performance). Instead, the protocol was structured to characterize stimulation-aware tremor dynamics, quantify modeling utility during intervention, and prospectively test whether model-informed parameter selection could improve suppression relative to reference conditions in a pilot setting. Accordingly, the present results should be interpreted as enabling evidence for systematic personalization and future data-driven closed-loop studies, rather than direct clinical-benefit claims.

Additional limitations include the relatively small and homogeneous cohort (*n* = 6) and the present focus on a single antagonist muscle pair (FCR/ECR), which may not fully capture the diversity of tremor phenotypes or multi-DOF tremor mechanisms encountered in broader clinical populations. Future work will therefore require larger endpoint-driven studies with standardized functional and clinical outcome measures, prospective assessment of tolerability and safety, and extension to multi-site ultrasound sensing for richer multi-muscle characterization. From a translational perspective, further progress will also depend on wearable and embedded implementations that reduce the bulkiness and tethering constraints of conventional ultrasound; our recent dual-array wearable ultrasound study provides an initial step in that direction [58].

## VI. CONCLUSION

This work presents an ultrasound-informed, stimulation-aware data-driven framework for modeling wrist tremor dynamics under sPNS. Ultrasound improved prediction accuracy over sPNS-only modeling in both the time and frequency domains, and the adaptive online-update model achieved the best overall performance. The learned model also identified ultrasound-derived muscle displacement and intermuscular latency as dominant contributors to tremor dynamics, providing physiologically meaningful insight for tremor personalization. In a pilot human-validation study, the model-informed protocols produced the strongest tremor suppression in one PD participant and one ET participant among the tested conditions. Together, these results support ultrasound-informed modeling for tremor characterization and provide an initial basis for model-guided personalization and future closed-loop neuromodulation studies.

## Supplementary Material

supp1-3709711
