# Supplementary material for: Ultrasound-Informed State Estimation of Wrist Tremor Dynamics via Koopman Operator for Personalized Sensory Peripheral Nerve Stimulation
Source: IEEE Trans Neural Syst Rehabil Eng. Author manuscript; Available in PMC 2026 Sep 1. (PMC13533337; doi:10.1109/TNSRE.2026.3709711)
Supplement: Supplementary file 1 [file NIHMS2196889-supplement-supp1-3709711.pdf]

Supplementary Materials

Xiangming Xue, Vidisha Ganesh, Daniel Roque, Tanya P. Garcia, Xiaoning Jiang *Fellow, IEEE*, and Nitin Sharma *Senior Member, IEEE*

S1. Koopman Operator Framework and Linear Approximation

This section provides the theoretical derivation underlying the Koopman/EDMD formulation summarized in Section II of the main manuscript. The Koopman operator offers a means of analyzing nonlinear systems through a linear representation in a higher-dimensional function space [1].

$$x_{k+1} = f(x_k, u_k), \tag{S1}$$

where $x_k \in \mathbb{R}^n$ is the state (e.g., kinematic measurements) and $u_k \in \mathbb{R}^m$ denotes external inputs (such as ultrasound-derived features or stimulation parameters), and f is an unknown nonlinear mapping.

A lifted observable vector is defined as

$$\Psi(x_k, u_k) = \begin{bmatrix} x_k^\top & \phi(x_k)^\top & u_k^\top \end{bmatrix}^\top, \tag{S2}$$

where $\phi(x_k)$ contains nonlinear basis functions. The total dimension of this lifted state is $N_K = n + n_\phi + m$. In this augmented space, the Koopman operator $\mathcal{K}$ advances observables linearly:

$$\Psi_{k+1} = \mathcal{K}\Psi_k. \tag{S3}$$

Since $\mathcal{K}$ is infinite-dimensional in theory, a finite approximation $K \in \mathbb{R}^{N_K \times N_K}$ is computed from data, leading to

$$\Psi_{k+1} \approx K\Psi_k. \tag{S4}$$

Partitioning K according to states, lifted coordinates, and inputs gives:

$$\left[\begin{array}{c} x_{k+1} \\ \psi_{n_k}(x_{k+1}) \\ \hline u_{k+1} \end{array}\right] \approx \left[\begin{array}{cc|c} A_{11} & A_{12} & B_{11} \\ A_{21} & A_{22} & B_{21} \\ \hline * & * & * \end{array}\right] \left[\begin{array}{c} x_k \\ \psi_{n_k}(x_k) \\ \hline u_k \end{array}\right] \tag{S5}$$

The top two block rows define the finite-dimensional linear predictor:

$$z_{k+1} = Az_k + Bu_k, \tag{S6}$$

where $z_k = [\, x_k^\top, \phi(x_k)^\top \,]^\top$ stacks original states and lifted coordinates. Here, $A = \begin{bmatrix} A_{11} & A_{12} \\ A_{21} & A_{22} \end{bmatrix}$ models autonomous lifted dynamics, and $B = \begin{bmatrix} B_{11} \\ B_{21} \end{bmatrix}$ maps the effect of inputs.

To determine A and B, we use M snapshot pairs:

$$\begin{aligned} X &= [x_1, \ldots, x_M], \\ X^+ &= [x_1^+, \ldots, x_M^+], \\ U &= [u_1, \ldots, u_M], \end{aligned} \tag{S7}$$

where $x_k^+ = f(x_k, u_k)$, together with their lifted counterparts

$$\begin{aligned} X_{\text{lift}} &= [\Psi(x_1, u_1), \ldots, \Psi(x_M, u_M)], \\ X_{\text{lift}}^+ &= [\Psi(x_1^+, u_1^+), \ldots, \Psi(x_M^+, u_M^+)]. \end{aligned} \tag{S8}$$

The operator K is obtained by solving the least-squares problem

$$K = \arg\min_{\tilde{K}} \; \|X_{\text{lift}}^+ - \tilde{K}X_{\text{lift}}\|_F^2, \tag{S9}$$

with the closed-form solution

$$K = X_{\text{lift}}^+ X_{\text{lift}}^\top (X_{\text{lift}} X_{\text{lift}}^\top)^\dagger, \tag{S10}$$

where $(\cdot)^\dagger$ is the Moore–Penrose pseudoinverse. The matrices A and B are then extracted from the appropriate block rows of K. This yields a linear predictor in lifted coordinates that preserves nonlinear tremor dynamics while enabling frequency-domain analysis and feedback control design [2]–[6].

S2. Recursive Updates for Online Tremor Modeling

This section provides the recursive online-update formulation referenced in Section II of the main manuscript and used for adaptive tremor modeling. Since tremor dynamics vary over time, a static model may lose accuracy. To maintain predictive performance, we adopt a recursive least squares (RLS) approach that incrementally updates the Koopman operator whenever new data are collected [7]–[9].

Let $\Psi_k = \Psi(x_k, u_k)$ denote the lifted state at time k, with $\Psi_k^+ = \Psi(x_{k+1}, u_{k+1})$. In the batch formulation, the operator is estimated by

$$K = X_{\text{lift}}^+ X_{\text{lift}}^T (X_{\text{lift}} X_{\text{lift}}^T)^\dagger. \tag{S11}$$

Recomputing this expression after every new measurement is computationally expensive. Instead, we define

$$Q_M = X_{\text{lift}_M}^+ X_{\text{lift}_M}^T, \quad P_M = (X_{\text{lift}_M} X_{\text{lift}_M}^T)^\dagger, \tag{S12}$$

so that $K_M = Q_M P_M$. When a new pair $(\Psi_{M+1}, \Psi_{M+1}^+)$ arrives, P and K can be updated efficiently using the matrix inversion lemma [10]:

$$P_{M+1} = P_M - \gamma_{M+1} P_M \Psi_{M+1} \Psi_{M+1}^T P_M, \tag{S13}$$

$$\gamma_{M+1} = \frac{1}{1 + \Psi_{M+1}^T P_M \Psi_{M+1}}, \tag{S14}$$

$$K_{M+1} = K_M + \gamma_{M+1}\big(\Psi_{M+1}^+ - K_M\Psi_{M+1}\big)\Psi_{M+1}^T P_M. \tag{S15}$$

To discount older data, a forgetting factor $0 < \lambda \leq 1$ is introduced:

$$\min_{\tilde{K}} \sum_{k=1}^{M} \lambda^{M-k} \|\Psi_{k+1} - \tilde{K}\Psi_k\|_2^2, \tag{S16}$$

leading to the weighted update

$$K_{M+1} = K_M + \tilde{\gamma}_{M+1}\big(\Psi_{M+1}^+ - K_M\Psi_{M+1}\big)\Psi_{M+1}^T \hat{P}_M, \tag{S17}$$

with

$$\tilde{\gamma}_{M+1} = \frac{1}{1 + \Psi_{M+1}^T \hat{P}_M \Psi_{M+1}}, \quad \hat{P}_M = \tilde{P}_M/\lambda. \tag{S18}$$

For efficiency, updates are triggered only when the prediction error over a recent window exceeds a tolerance ε:

$$\|\bar{X}_M^T - \hat{X}_M^T\|_2^2 \geq \varepsilon. \tag{S19}$$

This selective update rule ensures that the model adapts when significant changes in tremor behavior occur, while avoiding unnecessary computations [2].

Prior work has established theoretical guarantees for the RLS-based Koopman update, including convergence and stability properties [11]. Here, we extend this line of research by evaluating its performance in experimental tremor modeling.

S3. Ultrasound-Based Displacement Estimation and Data-Driven Compensation of Ultrasound Discontinuities in Dual-Probe Acquisition

This section describes the ultrasound-processing framework used to support stimulation-aware tremor dynamics modeling. Ultrasound-based speckle tracking was first used to estimate tissue displacement from the FCR and ECR muscles, yielding a continuous physiological representation of local muscle activity during stimulation. Because the alternating dual-probe acquisition scheme introduced structured temporal gaps in these displacement signals, a data-driven compensation approach was then used to reconstruct continuous ultrasound trajectories. The first subsection describes displacement estimation via speckle tracking, the second presents the recursive gap-filling method, and the remaining subsections provide validation using continuous ground truth data and evaluation of tremor-feature preservation after compensation.

A. Ultrasound-Based Muscle Displacement Estimation via Speckle Tracking

Ultrasound-based speckle tracking was used to estimate muscle tissue displacement from sequential image frames, providing high-resolution motion data from the flexor and extensor muscle groups. This method offers a robust, stimulation-artifact-free alternative to EMG and has been validated in prior studies [12]. In the present work, displacement was used as a low-dimensional and physiologically interpretable representation of local muscle dynamics for stimulation-aware tremor modeling. Alternative ultrasound representations, including echo-derived features, may also be incorporated in future work, but displacement was selected here for consistency with the present sensing pipeline and prior validation studies.

B. Data-Driven Gap-Filling via Recursive Model Prediction

Before applying the data-driven tremor dynamics model, it is essential to address discontinuities in US data arising from the alternating dual-probe acquisition protocol. Since each probe collects data only during a portion of the acquisition cycle, the resulting US signals for the FCR and ECR muscles contain periodic temporal gaps. These discontinuities hinder continuous modeling by obscuring the full temporal evolution of muscle activity during tremor episodes. To overcome this, we employ a data-driven online update approach that reconstructs missing data segments in real time. While designed for alternating dual-probe acquisition, the proposed method is broadly applicable to other scenarios involving incomplete or lost physiological data, such as sensor dropout or communication failures in real-time systems.

US data were acquired using two probes triggered alternately by a real-time target machine at 500 Hz. Each probe captured five consecutive frames over a 5 ms interval, followed by a 5-ms idle period. After every 10 ms cycle, the system switched to the other probe, which then collected data from the antagonist muscle group. This alternating acquisition resulted in a repeating 20-ms cycle, during which each muscle was sampled for 5 ms and idle for 15 ms, creating structured data gaps in the timeline. For example, the FCR probe acquired data from 0 to 5 ms, followed by a 15 ms gap while the system idled and collected data from the ECR (10–15 ms). This acquisition scheme is illustrated in Figure S1.

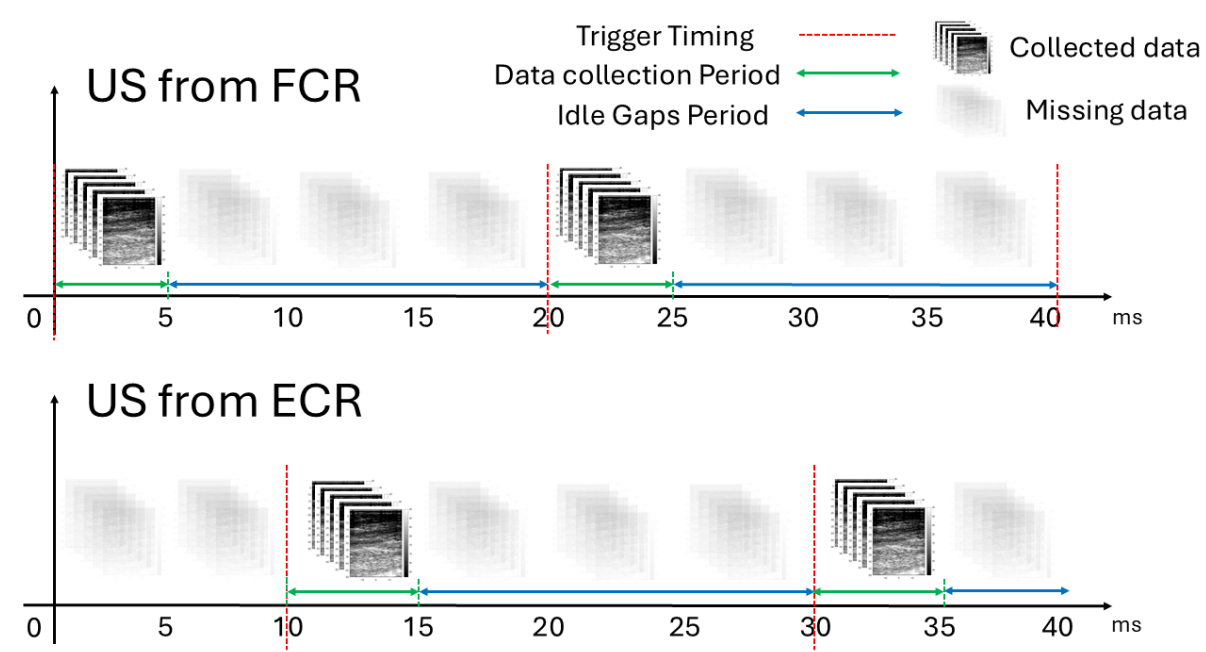

Fig. S1: Alternating acquisition protocol for US signals from the FCR and ECR muscles. Each probe collects 5 frames over a 5 ms interval (green arrows), with a 10 ms switching delay triggered by a red dashed line. This results in a 15 ms data gap for each muscle between successive acquisitions (blue arrows), producing discontinuous US signals that are addressed through data-driven gap-filling in subsequent modeling.

The alternating dual-probe acquisition was maintained throughout the 60-second recording, yielding approximately 15000 frames per muscle. Because each probe acquired data only 5 ms every 20 ms, the US timeline for each muscle included regular 15 ms gaps. To standardize the data for modeling, the frames were grouped into sets of five, forming 3000 frame groups per muscle. Tissue displacement was computed for all frames using the speckle tracking method described in [12], then assigned to a continuous timeline sampled at 1000 Hz to match the IMU. For model training and prediction, each group of five frames is divided into two sets: the first four frames are designated as the input data X_i, and the last four frames as the output data Y_i, for $i = 1 : 4$. Here, Y_i is the subsequent time step of X_i, i.e., $Y_i = X_{i+1}$. This forms 4 data pairs (X_i, Y_i) for both FCR and ECR in each group. These pairs serve as the training data for a data-driven modeling approach based on Extended Dynamic Mode Decomposition (EDMD) and Recursive Online Update Mechanisms. For each

group, the model $Y_i = A_i X_i$ is iteratively updated using the online recursive mechanism for $i = 1$ to $i = 4$, and then the model is used to predict the next 15 ms of data by applying $Y = A_4 X_i$ for $i = 5$ to $i = 20$, effectively filling the data gap in each group. The predicted data are then assigned to the placeholder positions in the continuous 1,000 Hz timeline, resulting in a reconstructed US dataset with no missing frames, ready for use in the tremor dynamics modeling process. The data processing for FCR and ECR gap fitting was accomplished through MATLAB (R2023b, MathWorks), and the data processing schematic is presented in Figure S2.

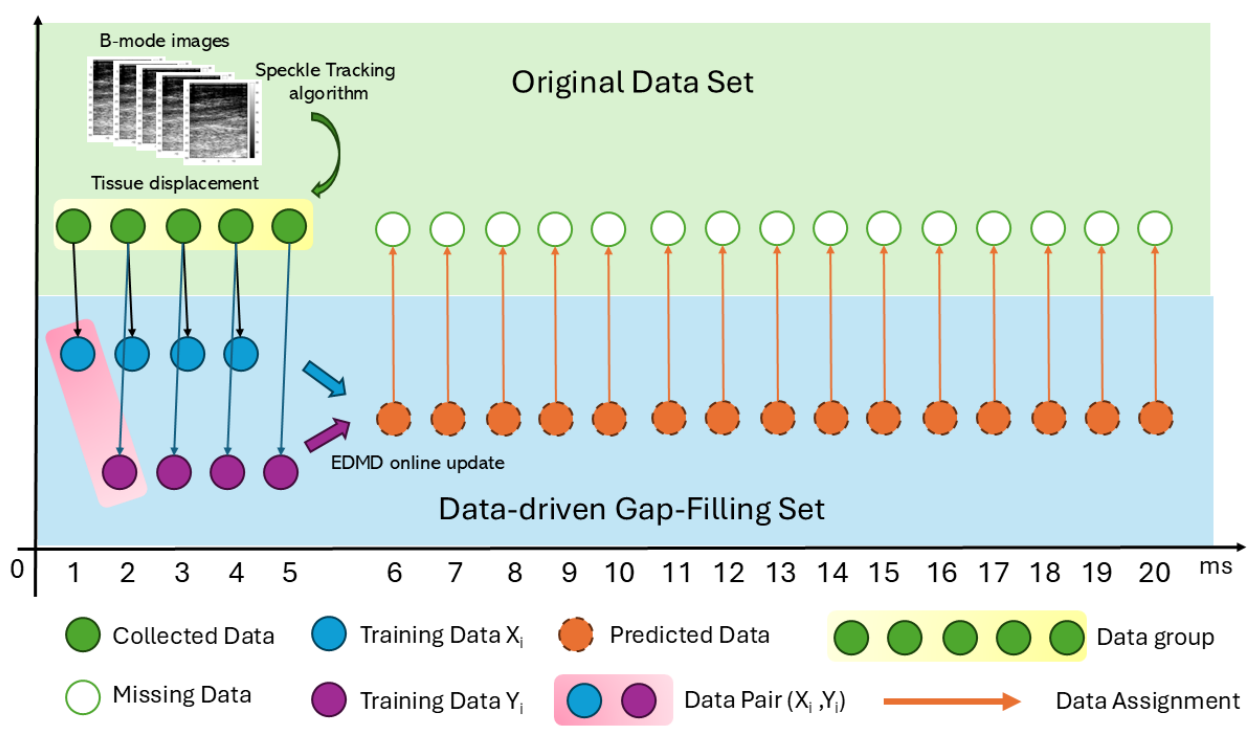

Fig. S2: Data-driven gap-filling process for US signals from FCR and ECR muscles. Frames are acquired for 5 ms every 20 ms, creating 15 ms data gaps. Tissue displacement is estimated via speckle tracking, and input-output pairs (X_i, Y_i) are used to train an EDMD model with online updates. The model predicts missing data to reconstruct a continuous 1,000 Hz signal for each muscle.

C. Validation under Continuous Ground Truth Acquisition

To validate the accuracy of the data-driven model used for gap-filling in the alternating US acquisition setup, we designed a separate validation experiment. The main challenge in validating the gap-filling approach is that there is no ground truth data available for one muscle (FCR or ECR) during the 15 ms data gap when the US system switches to the other probe. Therefore, we conducted an additional experiment using only one US probe to collect continuous US data, which served as the ground truth.

The experimental setup for this validation is similar to the one described in section II-C, with the key difference being the use of only one US probe. This probe continuously collected US data at a frame rate of 1000 Hz from the targeted muscle under sPNS, without alternating between muscles. After collecting the continuous data, we artificially introduced data gaps to mimic the structure of the dual-probe acquisition method. Specifically, we removed data points to create a 15 ms data gap for every 20 ms acquisition cycle, replicating the same temporal characteristics as the original dual-probe setup. This method allowed us to use the remaining continuous data as ground truth, while applying the data-driven model to fill in the artificially created gaps. By comparing the gap-filled data to the continuous, uninterrupted data, we were able to quantify the accuracy of the model's predictions. The experimental setup and validation process are illustrated in Figure S3.

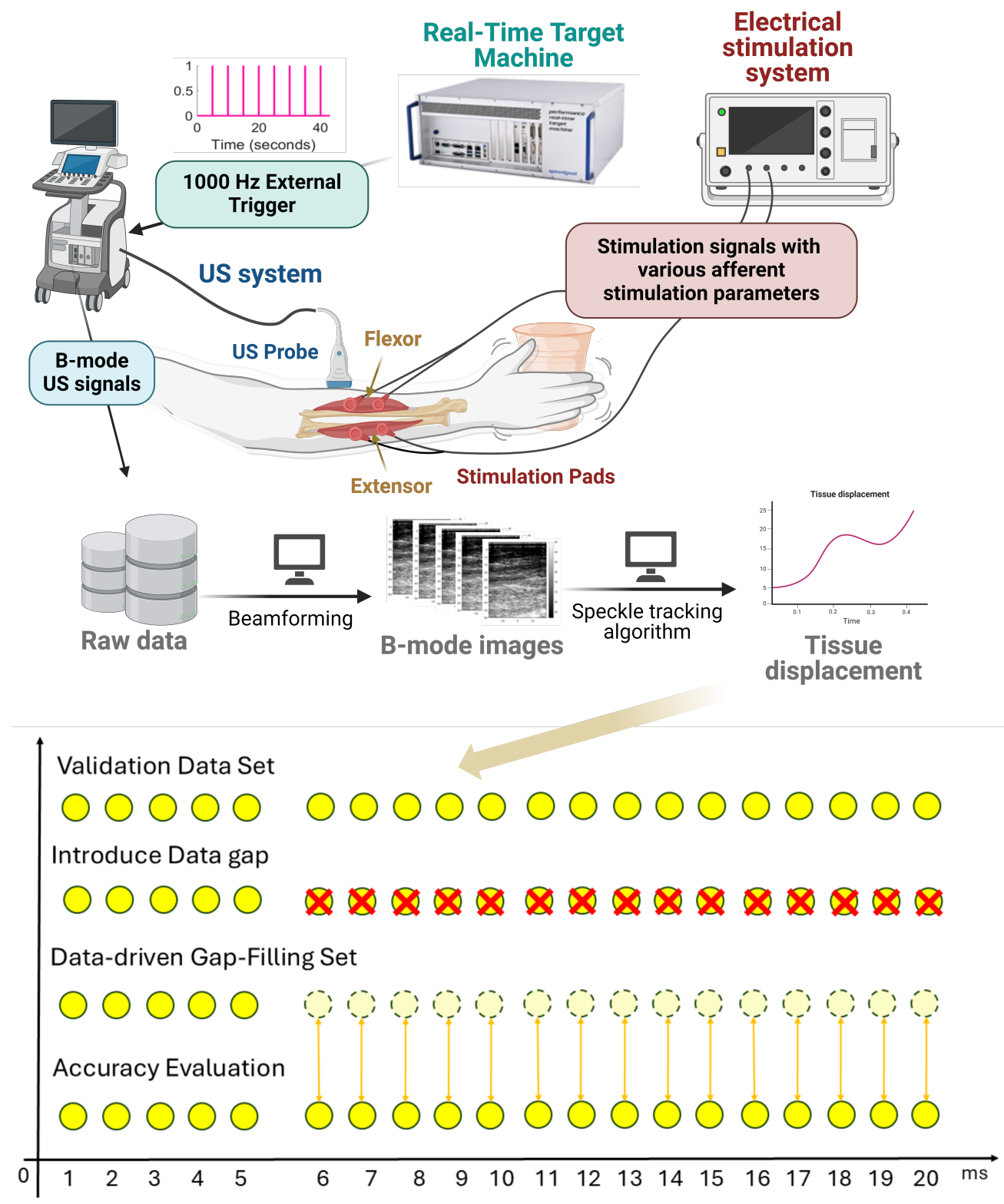

Fig. S3: Validation setup for evaluating the data-driven gap-filling method. Continuous US data were recorded at 1,000 Hz under sPNS, with artificial gaps introduced to simulate dual-probe acquisition. Gap-filled data were then compared to the original uninterrupted signal for accuracy assessment.

D. Preservation of Tremor Features in Discontinuous Ultrasound Data

We investigated the impact of data-driven interpolation for compensating temporal gaps inherent to dual-probe US acquisition setups, focusing on the preservation of key tremor features, dominant frequency and tremor power ratio (TPR). Figure S4 presents Bland–Altman analyses comparing the ground-truth (GT) and data-driven (DD) interpolated estimates for both the flexor and extensor muscle groups.

In terms of dominant frequency estimation for the flexor muscles, the DD approach exhibited a modest bias of -0.131 Hz, with limits of agreement (LoA) ranging from -1.271 to 1.009 Hz and an nRMSE of 15.9 %. For the extensor muscles, the bias was further reduced to -0.048 Hz, and LoA narrowed slightly to between -1.141 and 1.046 Hz, accompanied by an improved nRMSE of 14.5 %. Both muscle groups demonstrated minimal bias and symmetrical distributions around zero, confirming accurate frequency estimation by the data-driven interpolation approach.

Similar analysis for the tremor power ratio indicated an even stronger agreement. The flexor muscles showed a negligible bias of -0.015, with tight LoA of -0.160 to 0.131 and an

nRMSE of 14.0 %, demonstrating reliable preservation of tremor energy content. Extensor muscles displayed slightly greater variability, with a bias of -0.040 and LoA from -0.207 to 0.127, resulting in an nRMSE of 16.5 %. However, the overall bias remained small, and no systematic error was observed.

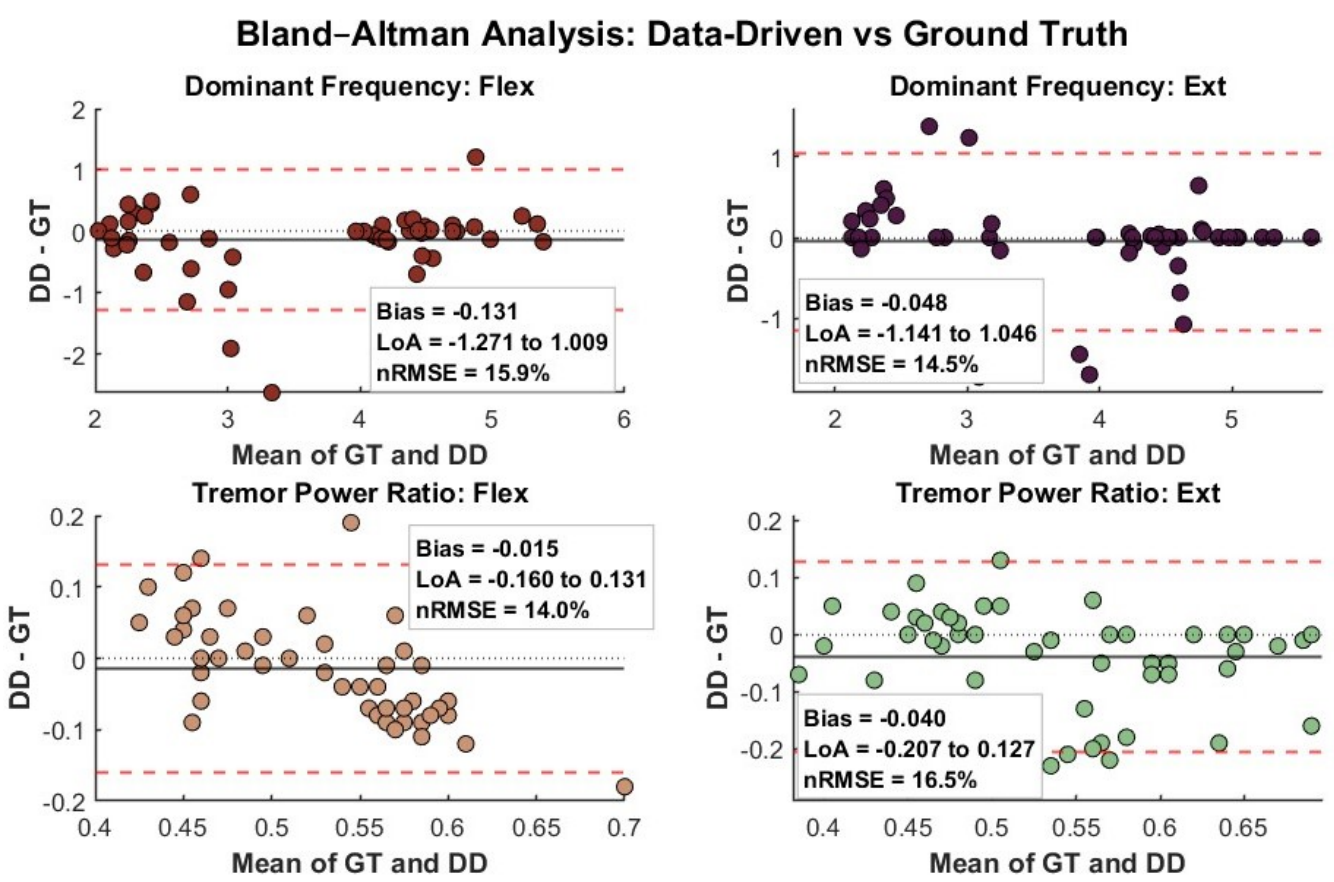

Fig. S4: Bland–Altman analysis comparing ground-truth (GT) and data-driven (DD) interpolation for dominant tremor frequency and tremor power ratio in flexor and extensor muscles. Solid lines represent the bias; dashed lines represent the 95 % limits of agreement (LoA).

E. Quantitative Validation Results under Continuous Ground Truth Acquisition

To evaluate the accuracy of our gap-filling strategy in the alternating US probe acquisition setup, we designed a separate validation experiment using continuous ground truth data. Unlike the dual-probe configuration—where one muscle's US data is intermittently missing—this experiment used a single US probe to acquire uninterrupted recordings from the Flexor Carpi Radialis (FCR) or Extensor Carpi Radialis (ECR) muscles. The data collected under this configuration allowed for a direct comparison between the true signal and the signal estimated by the data-driven (DD) model trained under simulated gap conditions.

Figure S5 presents the results of this validation. The left panel shows a Bland–Altman plot comparing DD-estimated versus ground truth dominant tremor frequencies. The mean bias was -0.050 Hz, with 95% limits of agreement ranging from -3.35 to 3.25 Hz. This suggests no consistent over- or underestimation of frequency by the DD model and acceptable agreement between true and predicted values.

The right panel displays a boxplot summarizing normalized root mean square error (nRMSE) for each muscle group. The ECR trials exhibited slightly lower variability and median error compared to the FCR trials, although both remained within a practically acceptable range. Across all patients and trials, the majority of frequency predictions fell within a 1 Hz deviation from the ground truth, confirming that the DD model preserves ET characteristics despite temporal signal discontinuities.

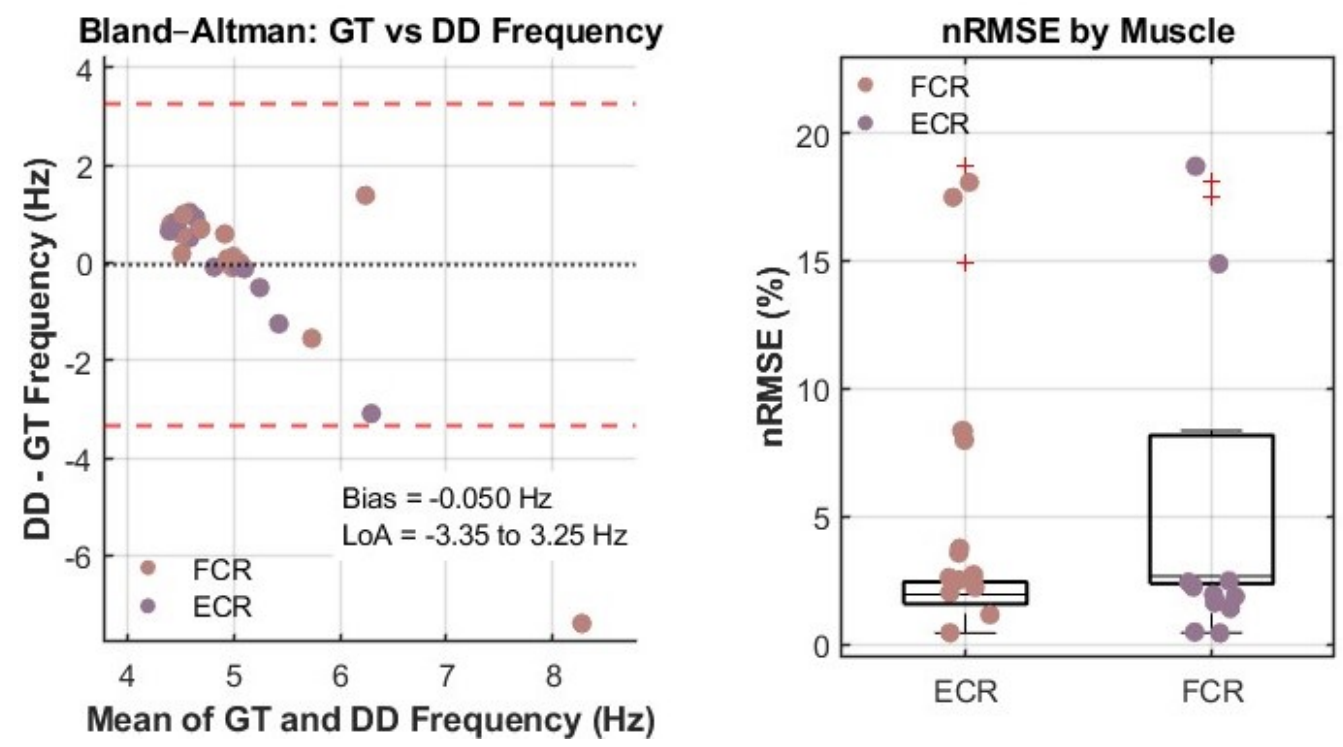

Fig. S5: Validation results using single-probe continuous US acquisition. **Left:** Bland–Altman plot showing agreement between ground truth (GT) and data-driven (DD) estimated dominant frequencies. **Right:** Boxplot of nRMSE by muscle group (FCR vs. ECR).

S4. Participant Characteristics and Ultrasound Imaging Results

This section provides supplementary participant information and ultrasound-based sensing results that support the experimental protocol described in the main manuscript. Specifically, we summarize participant demographic and clinical characteristics, show representative ultrasound imaging and tissue displacement examples from all participants.

A. Participant Demographic and Clinical Characteristics

The study cohort included four participants with Parkinson's disease (PD) and two participants with essential tremor (ET). Tremor severity was assessed using the Essential Tremor Rating Assessment Scale (TETRAS) for ET and the MDS-Unified Parkinson's Disease Rating Scale (MDS-UPDRS) Part 3 score for PD, as described in the main manuscript. Detailed demographic and clinical characteristics are summarized in Table SI.

B. B-Mode Ultrasound Images With ROI Across All Participants

To illustrate the ultrasound acquisition and ROI-selection procedure used for displacement tracking, we provide B-mode ultrasound images with the selected ROI across all participants in Fig. S6. These examples demonstrate that the imaging configuration and ROI-selection procedure were consistently applied across both the flexor and extensor muscle groups throughout the cohort.

C. Ultrasound-Derived Tissue Displacement and Wrist Kinematics Across All Participants

The corresponding ultrasound-derived tissue displacement and wrist kinematics across all participants are shown in Fig. S7. For each subject, the ultrasound signals from the FCR and ECR muscles are presented together with wrist kinematics measured by the IMU, illustrating the temporal relationship between local muscle motion and tremor behavior across the cohort.

TABLE SI: Demographic and Clinical Characteristics of Study Participants in Tremor Data Collection With sPNS

Participant ID	Tremor Disorder	Age	Gender	TETRAS Score	MDS-UPDRS Part 3 Score
T1	PD	67	Male	N/A	30
T3	ET	62	Male	22	N/A
T5	PD	62	Male	N/A	40
T9	PD	67	Female	N/A	29
T12	ET	69	Female	7.5	N/A
T13	PD	54	Male	N/A	36

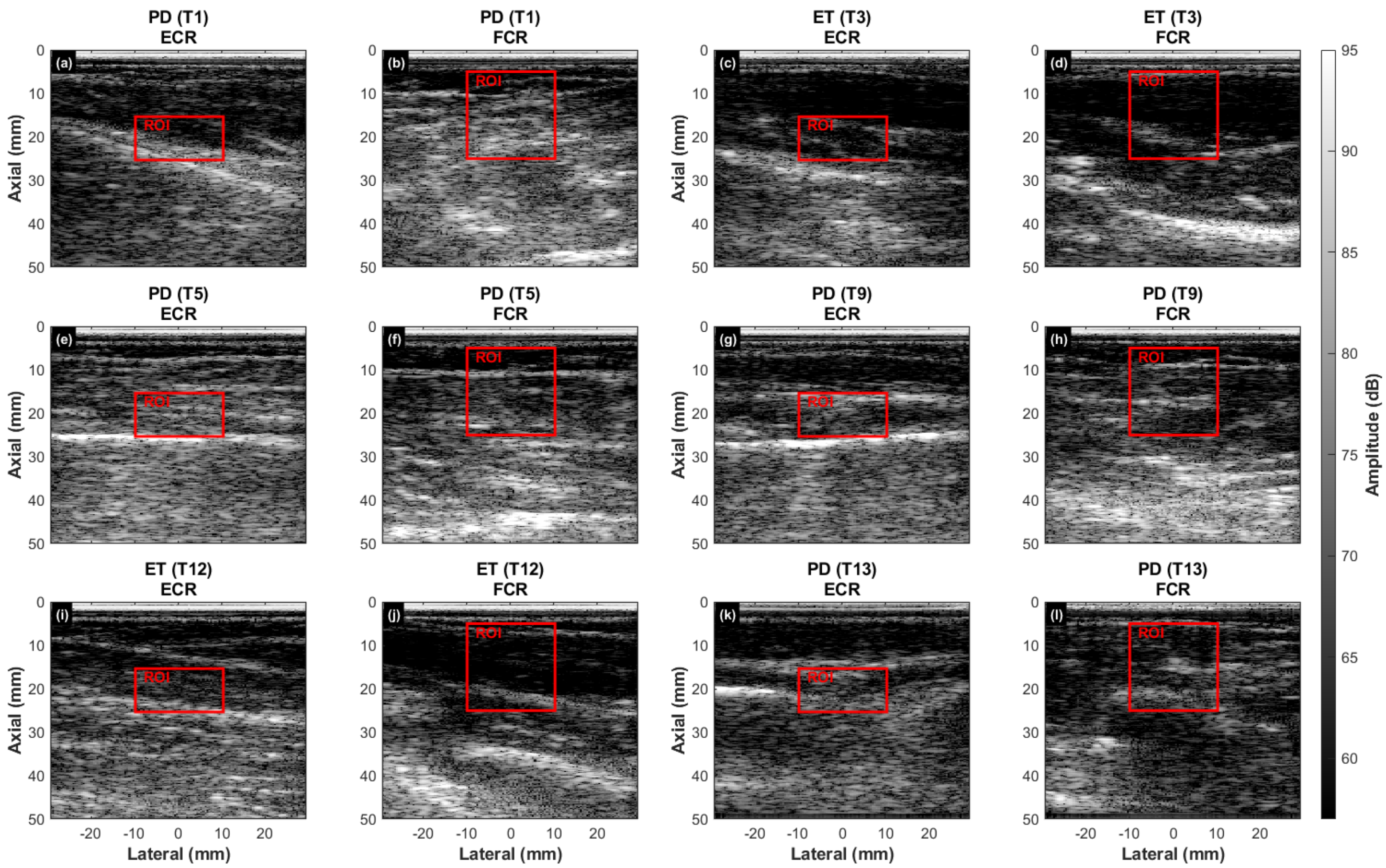

Fig. S6: B-mode ultrasound images with the selected ROI for displacement tracking across all participants. Panels show representative ECR and FCR imaging examples for PD (T1, T5, T9, and T13) and ET (T3 and T12). Red boxes indicate ROIs used for speckle tracking.

S5. Additional Model-Construction Details for Data-Driven Tremor Modeling

This section provides additional implementation details for the construction of the data-driven tremor models summarized in Section II-B of the main manuscript. Specifically, we describe signal preprocessing and synchronization across modalities, as well as the design and selection of observable functions used in the EDMD-based lifted representation.

A. Sensor Signal Processing

Data were preprocessed to ensure consistency and temporal alignment across the multi-sensor recordings collected from the ultrasound (US) and IMU sensors, together with the time-varying stimulation parameters. Because the experimental protocol involved multimodal sensing and sPNS inputs sampled at different rates, all signals were standardized to a common modeling time base prior to model training.

For the US data, tissue displacement was computed using the speckle-tracking procedure described in Supplementary Section S3. Because data were acquired alternately from two probes, the resulting discontinuities were first resolved using the data-driven gap-filling approach described in the same section, yielding continuous displacement signals at 1000 Hz. The reconstructed US signals were then downsampled to 100 Hz by averaging across non-overlapping 10-ms windows to match the modeling time base.

IMU signals were initially sampled at 1000 Hz and underwent linear detrending and outlier removal. The processed IMU signals were then resampled to 100 Hz to align temporally with the US data.

Stimulation parameters, including amplitude, burst frequency, and intermuscular latency, were originally sampled at lower rates. To synchronize them with the 100 Hz sensor data, cubic spline interpolation (MATLAB `interp1`, R2023b, MathWorks) was applied, producing smooth, time-aligned

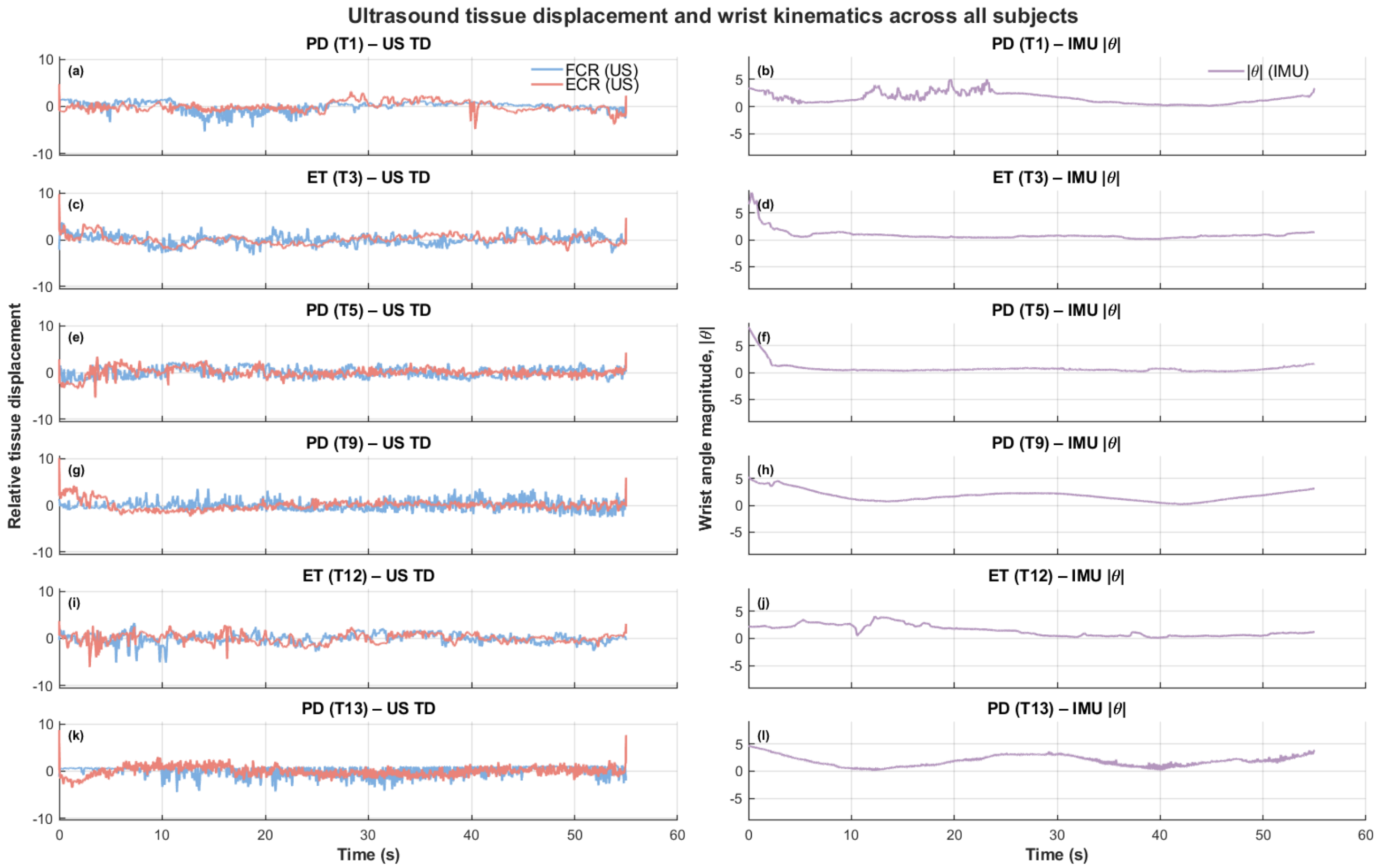

Fig. S7: Ultrasound-derived tissue displacement and wrist kinematics across all participants. For each subject, the upper panel shows relative tissue displacement from FCR and ECR measured by ultrasound, and the lower panel shows wrist angle magnitude $|\theta|$ measured by the IMU.

representations of the sPNS inputs for model construction.

B. Design and Selection of Observable Functions for Extended DMD

Our data-driven modeling approach uses extended dynamic mode decomposition (EDMD) [13] to approximate tremor dynamics under sPNS. In the EDMD framework, the observable function Ψ_k maps the original system states into a higher-dimensional feature space, enabling a lifted linear approximation of nonlinear dynamics.

The candidate observable functions evaluated in this study included original wrist-angle states and their temporal derivatives, harmonic terms, interaction terms, and polynomial terms, i.e.,

$$\Psi_k = \begin{bmatrix} x_k, \dot{x}_k, \ddot{x}_k \\ \text{Harmonic terms } (\sin(x_k), \cos(x_k), \dots) \\ \text{Interaction terms } (x_{ki} \times x_{kj}, \dots) \\ \text{Polynomial terms } (x_k^2, x_k^3, \dots) \end{bmatrix},$$

where x_k denotes the original wrist-angle states, and $\dot{x}_k$ and $\ddot{x}_k$ denote the corresponding velocity and acceleration terms. Harmonic terms were included to capture oscillatory tremor behavior, while polynomial and interaction terms were considered to account for potential nonlinear coupling in the dynamics.

To determine an effective observable-function combination, we systematically evaluated different subsets of these candidate observables by training EDMD models and comparing their prediction performance. Model performance was assessed using the normalized root mean square error (nRMSE) between predicted and true wrist-angle trajectories, and the observable combination associated with the lowest nRMSE was selected for the final model implementation.

S6. Supplementary Model Performance Results

This section provides supplementary results supporting the model-performance analyses presented in Section II-C of the main manuscript. Specifically, we report the full quantitative summary of time- and frequency-domain model accuracy across all participants, together with representative time-domain and frequency-domain examples that illustrate the differences among the three model variants.

A. Quantitative Summary of Model Accuracy Across Participants

Table SII summarizes the time-domain and frequency-domain performance of the three models across all six participants. Mean ± SD values are reported for time-domain nRMSE, dominant tremor frequency error, and tremor power ratio (TPR) error. This table provides the full numerical values corresponding to the cohort-level summary plots shown in Fig. 3 of the main manuscript.

	Time domain – nRMSE (%)			Frequency domain – Peak frequency (Error %)			Frequency domain – Tremor power ratio (Error %)		
Patient	*Model 1*	*Model 2*	*Model 3*	*Model 1*	*Model 2*	*Model 3*	*Model 1*	*Model 2*	*Model 3*
T1	35.20 ± 17.53	16.03 ± 3.36	0.29 ± 0.19	8.61 ± 5.78	8.46 ± 4.99	1.11 ± 2.49	61.18 ± 32.87	39.65 ± 25.09	0.95 ± 1.85
T3	31.42 ± 33.65	20.09 ± 7.30	0.06 ± 0.08	9.69 ± 6.36	8.19 ± 6.70	0.62 ± 2.24	53.67 ± 28.71	46.21 ± 25.47	0.87 ± 1.48
T5	28.24 ± 5.01	20.84 ± 3.36	0.17 ± 0.19	14.71 ± 7.19	14.12 ± 9.81	0.00 ± 0.00	34.01 ± 33.43	20.32 ± 24.45	0.49 ± 0.30
T9	33.14 ± 16.08	19.44 ± 5.33	0.04 ± 0.02	14.46 ± 8.98	12.67 ± 9.40	0.00 ± 0.00	16.65 ± 20.87	12.76 ± 14.75	0.82 ± 1.49
T12	34.57 ± 6.50	23.08 ± 3.87	0.07 ± 0.01	7.57 ± 5.11	7.56 ± 7.05	0.00 ± 0.00	43.60 ± 27.14	33.13 ± 31.29	2.77 ± 2.60
T13	48.50 ± 23.36	23.19 ± 5.17	0.22 ± 0.05	4.58 ± 3.47	4.42 ± 5.32	0.00 ± 0.00	35.76 ± 21.23	25.85 ± 15.46	0.03 ± 0.03

TABLE SII: Model performance across six participants in time- and frequency-domain metrics. Mean ± SD values are reported for three models under (i) time-domain nRMSE, (ii) frequency-domain dominant tremor frequency error, and (iii) frequency-domain tremor power ratio (TPR) error.

B. Representative Time-Domain Prediction Example

Figure S8 shows a representative trial from Participant T12, comparing predicted wrist-angle trajectories from the three models against ground truth. While Model 1 captures the general oscillation pattern but underestimates amplitude, Model 2 improves amplitude prediction and temporal tracking, and Model 3 most closely matches the observed trajectory throughout the trial.

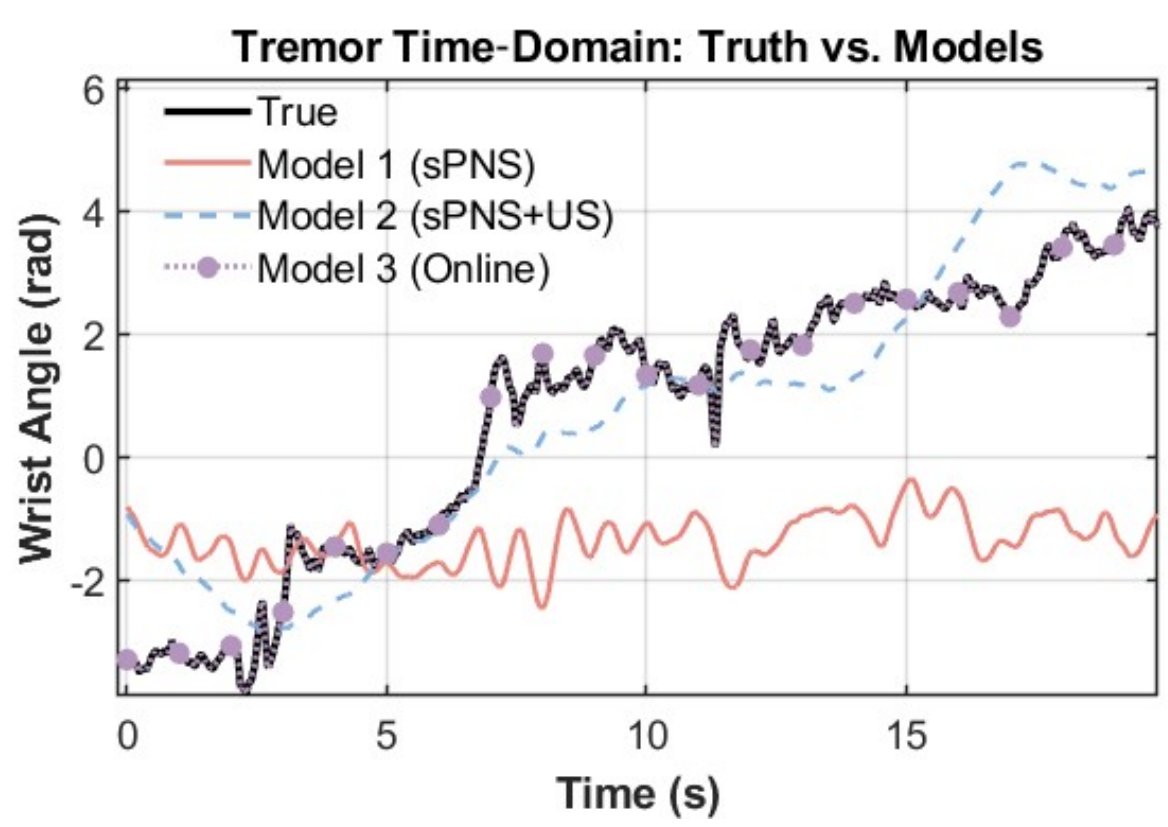

Fig. S8: Representative trial from Participant T12 showing predicted wrist angles from the three models against ground truth.

C. Representative Frequency-Domain Comparison

Figure S9 shows a representative spectral comparison from Participant T9. The spectrum predicted by Model 3 closely matches the ground-truth spectrum in both dominant-frequency location and tremor-band power, whereas Model 1 underestimates both spectral peak magnitude and overall tremor-band energy. This representative example complements the cohort-level frequency-domain summary shown in the main manuscript.

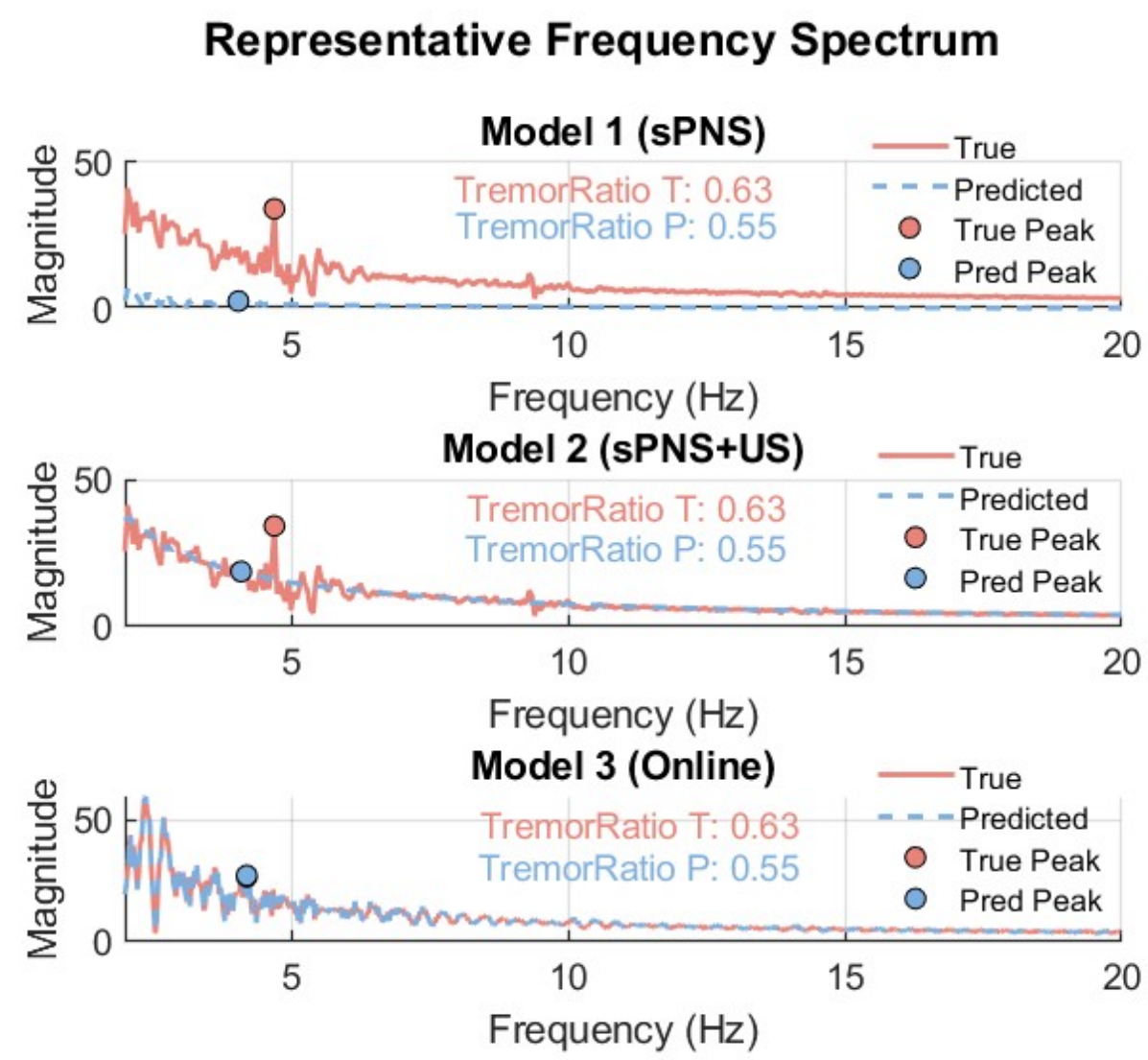

Fig. S9: Representative spectral comparison for Participant T9.

S7. Tremor Frequency Extraction from Model Spectral Properties

A. Significance of the Analysis

Accurate identification of dominant tremor frequency is essential for evaluating tremor severity and understanding its underlying physiological mechanisms. Conventional approaches primarily involve spectral analysis of externally measured sensor data, such as IMU signals. However, these techniques do not inherently reflect the system-level dynamics driving tremor oscillations. By contrast, our proposed approach directly utilizes the internal spectral characteristics of the state-space model, obtained through eigenvalue decomposition, to determine tremor frequencies. This methodology provides a direct, structured, and physiologically interpretable representation of the oscillatory dynamics governing tremor, circumventing limitations of traditional spectral methods.

B. Computational Implementation

Our approach employs eigenvalue decomposition of the learned system matrix A from the data-driven model to identify the dominant tremor frequencies. Specifically, eigenvalues are examined to isolate oscillatory modes within the tremor frequency range (4–12 Hz). Each eigenmode's contribution to tremor oscillation is quantified by combining eigenvectors and input matrix weights, providing an explicit frequency estimation associated with physiological tremor behavior. For each wrist state $(\theta_x, \theta_y, \theta_z)$, the dominant frequency is identified by

selecting the eigenmode with the highest weighted participation factor within the tremor frequency band. If no eigenmode lies within the defined band, the most influential eigenmode outside this range is selected as a secondary criterion.

C. Model Application

The described tremor frequency extraction methodology was applied to Model 2. Dominant tremor frequencies estimated from model spectral properties were compared with ground truth measurements obtained through conventional spectral analysis.

D. Model-Based Estimation of Tremor Frequencies

To evaluate the model's capability to estimate tremor frequency directly from learned system dynamics, we extracted the dominant frequency from the eigenvalues of the state transition matrix A, as described in Section S7. This model-derived frequency was compared to the ground truth frequency obtained via Fourier analysis of the IMU signals across multiple trials for each participant.

Figure S10 presents a box plot comparing the distribution of dominant tremor frequencies estimated from the data-driven model to the ground truth across six participants. Each box plot shows the spread of model-derived frequency estimates across trials, along with markers denoting the mean ground truth frequency (inverted black triangle) and model-estimated mean frequency (white triangle).

Quantitatively, the average percentage error across all participants was 16.1%. Participant-specific errors ranged from as low as 0.05 (T13) to 0.56 (T9), indicating strong agreement in most cases. For example, the model closely tracked the tremor frequency in T5 (mean ground truth: 5.04 Hz, model: 5.36 Hz), with a low mean error of 0.12. In contrast, larger discrepancies occurred in T3 and T9, where the frequency was occasionally overestimated, possibly due to secondary spectral peaks or non-stationary oscillations affecting eigenvalue interpretation.

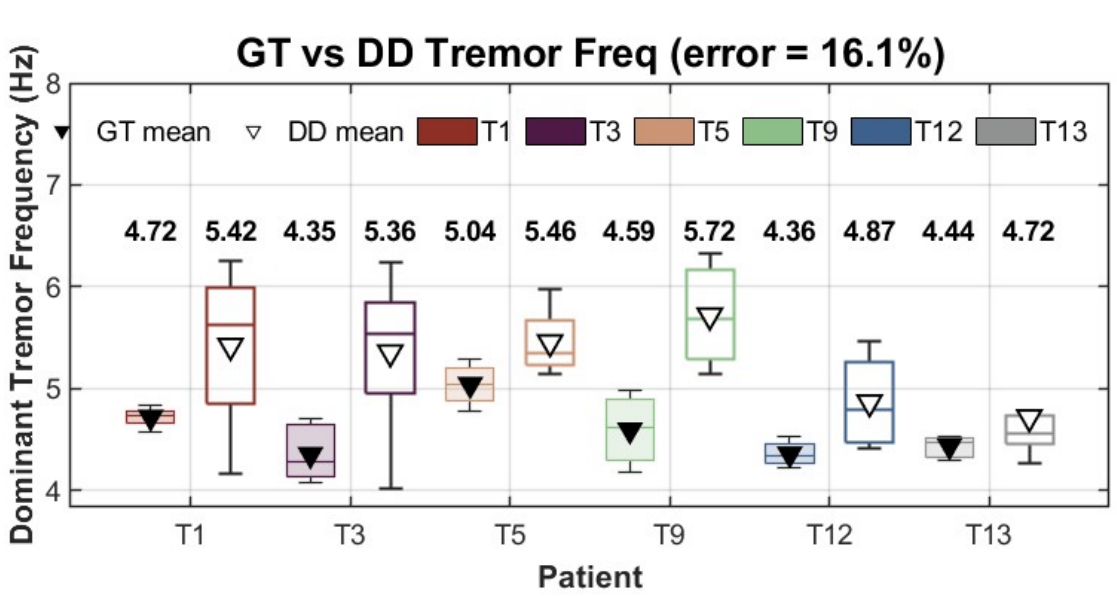

Fig. S10: Comparison of model-derived vs. ground truth dominant tremor frequencies for each participant. Box plots show the distribution of frequencies estimated from the model across all trials. Black inverted triangles indicate the mean ground truth frequency; white triangles indicate the mean model-estimated frequency.

S8. COMPUTATION OF INPUT MODAL CONTRIBUTIONS (CGSVD)

To systematically assess the relative influence of different inputs (sPNS parameters and ultrasound features), we employed a controllability-based subspace analysis on the identified Koopman models. Specifically, the controllability Gramian was approximated and decomposed via singular value decomposition (SVD), and each input was projected onto the dominant controllable directions. The normalized projection magnitudes provide a percentage breakdown of the contribution of each input channel to the overall system controllability. The full algorithmic procedure is summarized in Algorithm S1.

Algorithm S1 Controllability Gramian & SVD Decomposition (CGSVD) for Input Dominance

Inputs:

$$A \in \mathbb{R}^{n\times n}, \quad B \in \mathbb{R}^{n\times m}, \quad K \in \mathbb{N}$$

Output: Dominant input percentages $\{\pi_i\}_{i=1}^{m}$.

label=1.

1) **Build (approximate) controllability Gramian**

$$W_c \;=\; \sum_{k=0}^{K} A^k\, B\, B^\top\, (A^k)^\top$$

by accumulating $A^k B B^\top (A^k)^\top$ in a loop (e.g. $K = 100$).

2) **Extract the dominant directions via SVD**

$$[U, S, V] \;=\; \mathrm{svd}\big(W_c\big)$$

The columns of U are the principal "reachability" modes, ordered by singular value.

3) **Project each input onto those modes**

$$P \;=\; \big|B^\top U\big|, \quad P_{ij} \;=\; \text{strength of input } i \text{ on mode } j.$$

4) **Sum and normalize**

$$\text{influence}_i \;=\; \sum_{j=1}^{n} P_{ij}, \qquad \pi_i \;=\; 100\times\frac{\text{influence}_i}{\sum_{p=1}^{m}\text{influence}_p}$$

which yields a percentage breakdown of how much each input "dominates" the system's controllability.

A. Method Application

The input-dominance analysis was conducted using Model 2 from the main manuscript, which incorporates both sPNS and US inputs. To isolate the contribution of different input groups, three configurations were considered: (1) an sPNS-only configuration using matrices A and B, (2) a US-only configuration using matrices A and H, and (3) a combined configuration using A together with the aggregated input matrix $[B \quad H]$. In the main manuscript, the sPNS-only result is shown because it directly motivates the dominance-guided prioritization of stimulation parameters in the protocol-selection framework.

The supplementary figures below provide the additional US-only and combined input-dominance results.

B. Additional Results: Ultrasound-Only and Combined Input Dominance

Figure S11 shows the distribution of input dominance between FCR and ECR muscle displacement measurements in the US-only configuration. Participants displayed substantial variability in dominance distribution between muscles: the FCR muscle dominated for participants T1, T3, and T12, whereas the ECR muscle showed stronger dominance for participants T5, T9, and T13. This result supports the view that subject-specific tremor expression may be associated with different antagonist muscles and motivates individualized consideration of ultrasound sensing locations.

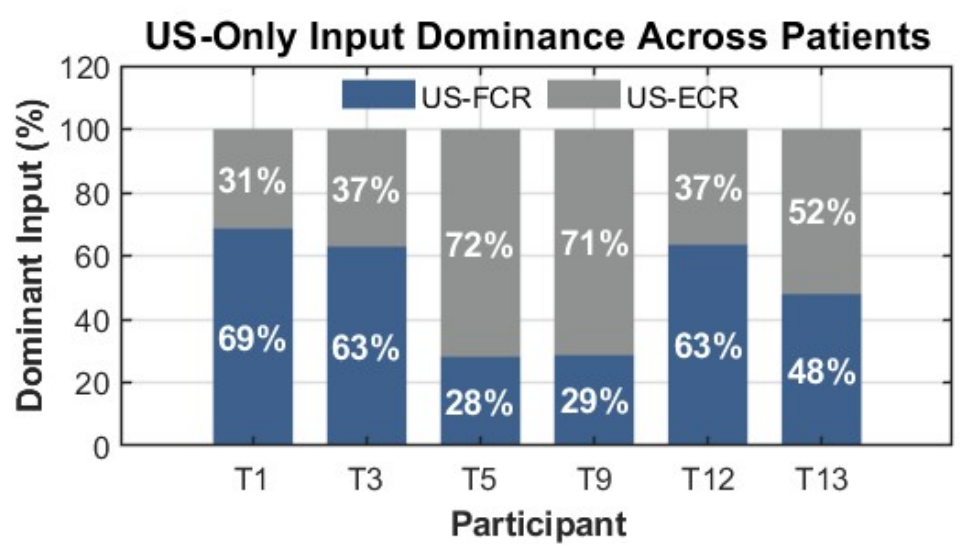

Fig. S11: Dominance of US inputs (FCR vs. ECR muscle displacement) for each participant, highlighting muscle-specific variability.

Figure S12 shows the combined analysis of sPNS and US inputs. In this configuration, the two ultrasound-derived inputs (US-FCR and US-ECR) exhibited substantially larger dominance than most sPNS parameters, while the intermuscular delay parameter retained a strong contribution relative to the remaining stimulation inputs. This result complements the main-manuscript finding that stimulation delay is the most influential parameter within the sPNS subset and further shows that direct physiological sensing contributes strongly to the controllability structure of the learned tremor dynamics.

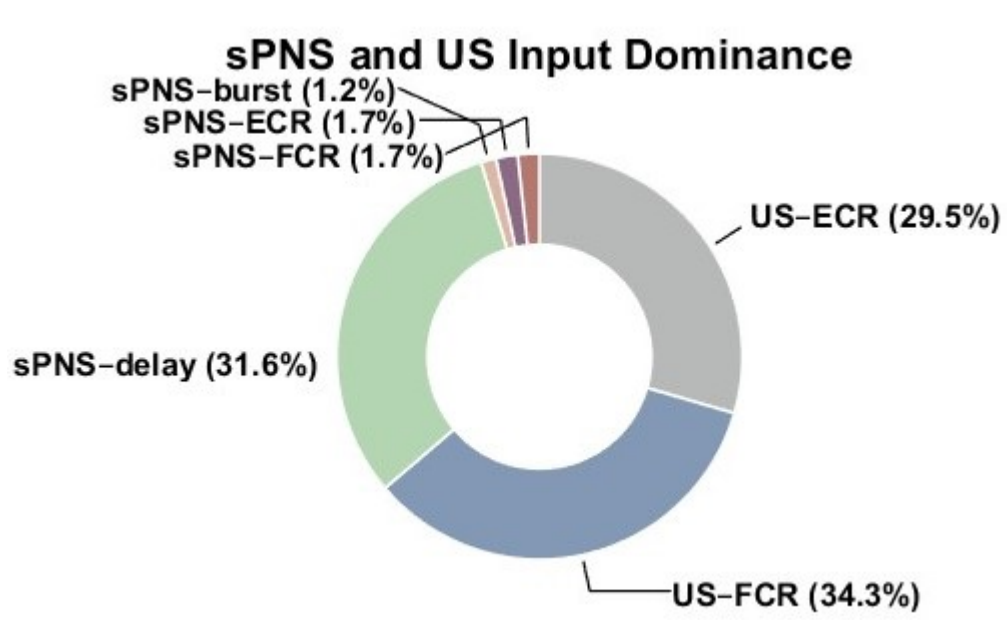

Fig. S12: Combined analysis of sPNS and US input dominance. US-derived inputs (FCR and ECR) and sPNS-delay show the highest dominance, underscoring their critical roles in tremor prediction.

S9. Identification of Dominant Tremor States

The core methodology evaluates dominant state contributions based on a combination of system dynamics and energy distribution across angular states. First, eigen-decomposition is performed on the transition matrix A, and both left and right eigenvectors are extracted. A weighted modal contribution is computed for each state using the interaction between these eigenvectors and the input matrix B. This contribution is then compared to the signal-level power observed in both true and predicted wrist angle data. The algorithm is summarized as Algorithm S2.

Algorithm S2 Modal Decomposition & Participation Factor Analysis (MDPFA) for Tremor State Dominance

Inputs:

$$A \in \mathbb{R}^{n\times n}, \quad B \in \mathbb{R}^{n\times m}, \quad x_{\text{true}} \in \mathbb{R}^{3\times T}$$

Outputs:

$$\{\pi_i\}_{i=1}^3, \quad \{\pi_{\text{true},i}\}_{i=1}^3, \quad \{\text{Error}_i\}_{i=1}^3$$

label=2.

1) **Eigen-decomposition:**

$$(V, D) \leftarrow \text{eig}(A), \quad W \leftarrow V^{-1}.$$

2) **Mode contributions:** For each state $i = 1, 2, 3$ and each mode $j = 1, \dots, n$,

$$C_{ij} = |V_{ij}|\,|W_{ji}|\,\big\|W_{j,:}\,B\big\|.$$

3) **Model-level normalization:**

$$c_i = \sum_{j=1}^{n} C_{ij}, \quad \pi_{\text{model},i} = 100\,\frac{c_i}{\sum_{k=1}^{3} c_k}.$$

4) **Signal-level percentages:**

$$\pi_{\text{true},i} = \frac{\sum_t x_{\text{true},i}(t)^2}{\sum_{p=1}^{3}\sum_t x_{\text{true},p}(t)^2}.$$

5) **Error metric:**

$$\pi_{\text{error},i} = \big|\pi_{\text{model},i} - \pi_{\text{true},i}\big|.$$

A. Method Application

The dominant tremor state analysis was applied to Model 2, which incorporates both sPNS and US-derived displacement inputs. Modal contributions for each angular state (θ_x, θ_y, and θ_z) were computed using the model-based procedure above and compared against signal-based contributions derived from the corresponding ground-truth wrist-angle trajectories. This comparison was used to evaluate whether the model's internal modal structure was consistent with the observed axis-level tremor behavior.

B. Results

Table SIII reports the state-dominance prediction errors across all six participants. The error metric $\pi_{\text{error},i}$ quantifies

the deviation between model-derived and ground-truth dominance percentages for each wrist-angle state. Across participants, the average errors were comparable for θ_x, θ_y, and θ_z, indicating that the model did not exhibit a strong systematic bias toward any one axis overall. However, subject-specific variability was evident. For example, Participant T5 exhibited larger error in the θ_y direction than in the other two axes, whereas Participant T13 showed relatively larger error for θ_z. These results suggest that the relative contribution of wrist-angle states to tremor expression differs across participants and further support the need for individualized state interpretation when using the learned model for tremor characterization and control-related analysis.

TABLE SIII: State-dominance prediction errors (%) across wrist angles and participants.

Patients	$\pi_{\text{error},i}(\%)$			
	**wrist x**	**wrist y**	**wrist z**	Average across patients
T1	20.16	12.20	15.78	16.05
T3	13.88	14.22	14.24	14.11
T5	33.92	45.99	22.71	34.21
T9	29.08	25.84	29.68	28.20
T12	20.41	17.76	16.27	18.15
T13	14.46	13.66	23.35	17.16
Average across wrist angle	21.99	21.61	20.34	21.31

S10. Protocol-Evaluation Objective and Candidate Ranking

For protocol selection, each candidate stimulation setting was evaluated using the subject-specific prediction model described in the main manuscript. Let $X^{(c)}$ denote the model-predicted wrist-angle trajectory for candidate protocol c, and let $x_k^{(c)}$, $y_k^{(c)}$, and $z_k^{(c)}$ denote the predicted wrist-angle states at prediction step k along the three axes. In the present implementation, the predicted wrist-motion magnitude trajectory was defined as

$$m_k^{(c)} = \sqrt{\left(x_k^{(c)}\right)^2 + \left(y_k^{(c)}\right)^2 + \left(z_k^{(c)}\right)^2}.$$

The protocol-evaluation objective was then defined as the root-mean-square (RMS) value of the predicted three-axis wrist-motion magnitude over the prediction horizon:

$$J^{(c)} = \sqrt{\frac{1}{N_{\text{roll}}} \sum_{k=1}^{N_{\text{roll}}} \left(m_k^{(c)}\right)^2},$$

where N_{roll} is the number of prediction samples in the simulation window. Thus, lower values of $J^{(c)}$ correspond to lower predicted wrist-motion magnitude and, therefore, stronger predicted suppression.

Candidate protocols were ranked according to $J^{(c)}$, and the selected subject-specific protocol was

$$c^* = \arg\min_{c \in \mathcal{C}} J^{(c)},$$

where $\mathcal{C}$ denotes the predefined candidate protocol set. In addition to the top-ranked candidate, nearby highly ranked candidates were retained as backup options for prospective testing.

In the present implementation, the candidate set was defined with stimulation amplitude fixed at 4 mA, burst frequency searched over $\{2, 3, 4, \ldots, 12\}$ Hz, and intermuscular onset latency searched over $\{0, 0.01, 0.02, \ldots, 0.25\}$ s, consistent with the dominance-guided prioritization strategy described in the main text.

S11. Representative Window-Aligned Comparisons for Human Validation

To complement the condition-level suppression ratios reported in Section IV-C of the main manuscript, we provide representative window-aligned comparisons of wrist angular-velocity magnitude for the tested conditions in the two pilot participants. These comparisons are now shown in the same RMS-based framework used for the human-validation analysis.

For each displayed trial, the gyroscope signals were detrended and converted to the three-axis angular-velocity magnitude. The curves shown in Fig. S13 represent the normalized moving RMS of this signal in the initial and later protocol windows, while the reported suppression values correspond to the window-level suppression ratio defined from the RMS angular velocity in the main manuscript.

For T9, the model-prescribed protocol C2 showed the clearest reduction in wrist angular-velocity magnitude relative to the reference window, consistent with the condition-level suppression summary in the main text. The continuous reference condition C1 yielded only modest benefit, whereas the delay-perturbed condition C3 showed partial improvement but remained inferior to C2 in the representative comparison.

For T12, the contrast among protocols was more pronounced. The continuous condition C1 remained ineffective, whereas the model-prescribed condition C2 produced the strongest reduction in wrist angular-velocity magnitude. The delay-perturbed condition C3 preserved some suppression effect, but remained less effective than C2 in the representative example.

These representative window-aligned comparisons are consistent with the RMS angular-velocity suppression ratios reported in the main text and further support the interpretation that the model-informed protocol produced the strongest suppression in both pilot cases.

References

[1] B. O. Koopman, "Hamiltonian systems and transformation in hilbert space," *Proceedings of the national academy of sciences of the united states of america*, vol. 17, no. 5, p. 315, 1931.

[2] X. Xue, A. Iyer, and N. Sharma, "Koopman-based data-driven model predictive control of limb tremor dynamics with online model updating: A theoretical modeling and simulation approach," in *2023 American Control Conference (ACC)*, pp. 2873–2878, IEEE, 2023.

[3] M. Korda and I. Mezić, "Linear predictors for nonlinear dynamical systems: Koopman operator meets model predictive control," *Automatica*, vol. 93, pp. 149–160, 2018.

[4] E. Kaiser, J. N. Kutz, and S. L. Brunton, "Data-driven approximations of dynamical systems operators for control," in *The Koopman Operator in Systems and Control*, pp. 197–234, Springer, 2020.

[5] S. Klus, F. Nüske, S. Peitz, J.-H. Niemann, C. Clementi, and C. Schütte, "Data-driven approximation of the koopman generator: Model reduction, system identification, and control," *Physica D: Nonlinear Phenomena*, vol. 406, p. 132416, 2020.

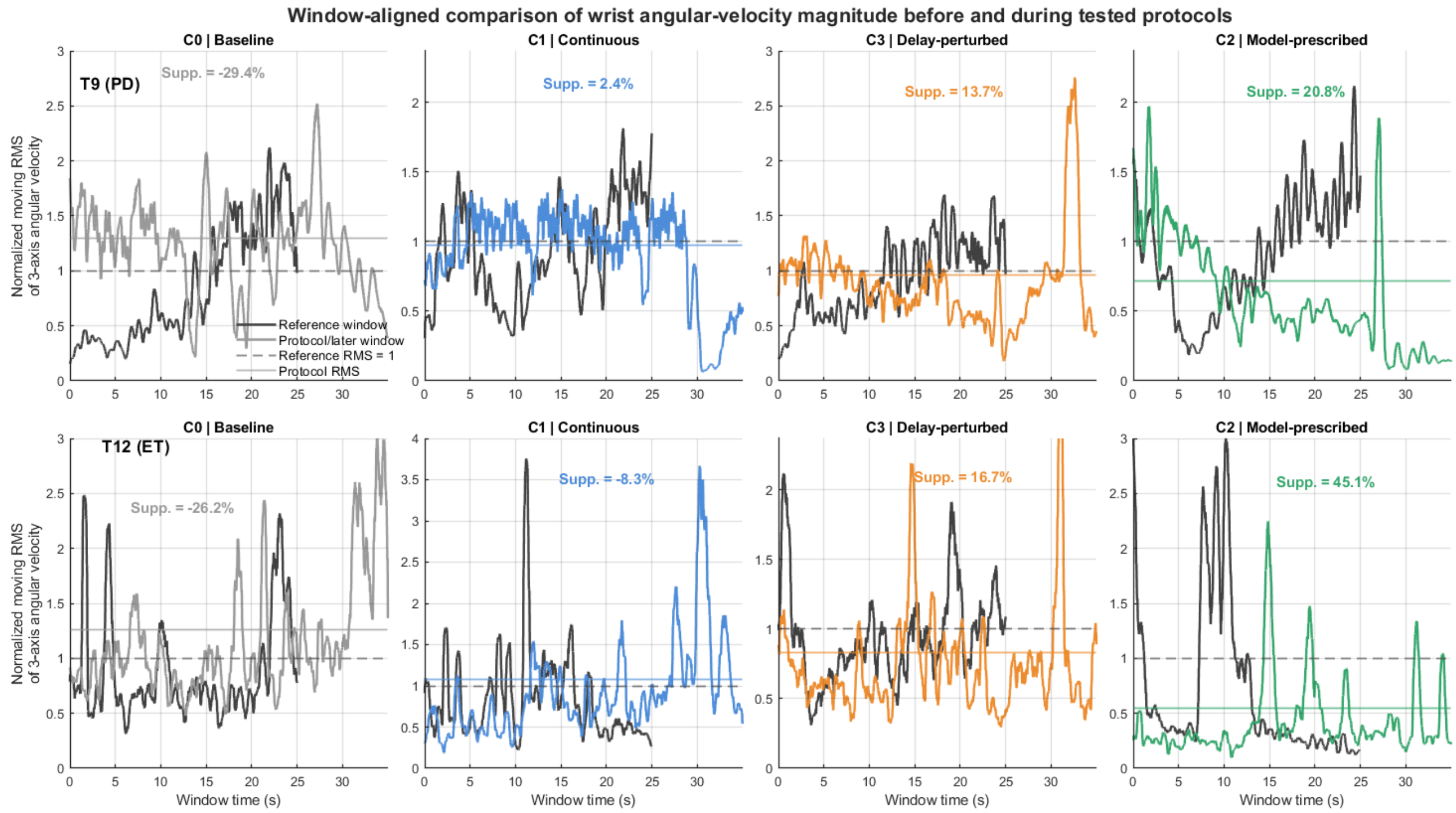

Fig. S13: Representative window-aligned comparisons of normalized moving RMS of the three-axis angular-velocity magnitude before and during the tested protocols for T9 and T12. Black traces denote the reference window, colored traces denote the later protocol window, and horizontal lines indicate the corresponding RMS levels used to compute suppression. In both participants, the model-prescribed condition C2 produced the largest reduction in RMS angular velocity among the tested stimulation conditions shown here, whereas C1 remained weak or ineffective and C3 showed intermediate benefit.

[6] J. L. Proctor, S. L. Brunton, and J. N. Kutz, "Generalizing koopman theory to allow for inputs and control," *SIAM Journal on Applied Dynamical Systems*, vol. 17, no. 1, pp. 909–930, 2018.
[7] T. C. Hsia, *System identification: least-squares methods*. Free Press, 1977.
[8] H. M. Calderón, E. Schulz, T. Oehlschlägel, and H. Werner, "Koopman operator-based model predictive control with recursive online update," in *2021 European Control Conference (ECC)*, pp. 1543–1549, IEEE, 2021.
[9] H. Zhang, C. W. Rowley, E. A. Deem, and L. N. Cattafesta, "Online dynamic mode decomposition for time-varying systems," *SIAM Journal on Applied Dynamical Systems*, vol. 18, no. 3, pp. 1586–1609, 2019.
[10] K. J. Åström and B. Wittenmark, *Adaptive control*. Courier Corporation, 2013.
[11] X. Xue, A. Iyer, D. Roque, and N. Sharma, "Nonlinear system identification of tremors dynamics: A data-driven approximation using koopman operator theory," in *2023 11th International IEEE/EMBS Conference on Neural Engineering (NER)*, pp. 1–4, IEEE, 2023.
[12] A. Iyer, X. Xue, V. Ganesh, X. Jiang, C. Gallippi, D. Roque, and N. Sharma, "Ultrasound imaging triggered tremor suppression with personalized afferent stimulation frequency," *IEEE Transactions on Biomedical Engineering*, 2024.
[13] M. O. Williams, I. G. Kevrekidis, and C. W. Rowley, "A data–driven approximation of the koopman operator: Extending dynamic mode decomposition," *Journal of Nonlinear Science*, vol. 25, no. 6, pp. 1307–1346, 2015.
